# Mobile Health Fitness Interventions: Impact of Features on Routine Use and Data Sharing Acceptability

**DOI:** 10.1016/j.jacadv.2023.100613

**Published:** 2023-09-22

**Authors:** Amir Razaghizad, Turney McKee, Isabelle Malhamé, Matthias G. Friedrich, Nadia Giannetti, Andrew Coristine, Anders Johnson, Euan A. Ashley, Steven G. Hershman, Brooke Struck, Sekoul Krastev, Dan Pilat, Abhinav Sharma

**Affiliations:** a Centre for Outcomes Research and Evaluation, Research Institute of the McGill University Health Centre, Montreal, Quebec, Canada; b DREAM-CV Lab, McGill University Health Centre, McGill University, Montreal, Quebec, Canada; c Division of Cardiology, McGill University Health Centre, McGill University, Montreal, Quebec, Canada; d The Decision Lab, Montreal, Quebec, Canada; e Division of General Internal Medicine, Department of Medicine, McGill University Health Centre, Montreal, Quebec, Canada; f Courtois Cardiovascular Signature Program, Research Institute of the McGill University Health Centre, Montreal, Quebec, Canada; g Division of Cardiovascular Medicine, Department of Medicine, Stanford University, Stanford, California, USA

**Keywords:** adherence, data sharing, health technology, mobile health, physical activity, primary prevention, public health, smartphone applications

## Abstract

**Background:**

Mobile health (mHealth) interventions are increasingly being used for cardiovascular research and physical activity promotion.

**Objectives:**

As a result, the authors aimed to evaluate which features facilitate and impede routine engagement with mobile fitness applications.

**Methods:**

We distributed a pan-Canadian online questionnaire via the behavioral research platform Prolific.co to evaluate what features are associated with the use and routine engagement (ie, daily or weekly use) of mHealth fitness applications and attitudes about data sharing. Binary logistic regression was used to quantify the association between these endpoints and exploratory factors such as the perceived utility of various mHealth application features.

**Results:**

The survey received 694 responses. Most people were women (62%), the median age was 28 years (range: 18-78 years), and most people reported current use of an mHealth fitness application (48%). The perceived importance of personal health (OR: 2.40; 95% CI: 1.34-4.50) was the factor most associated with the current use of an mHealth fitness application. The feature most associated with routine engagement was the ability to track progress toward a goal (OR: 5.10; 95% CI: 2.73-9.61) while the most significant barrier was the absence of goal customization features (OR: 0.44; 95% CI: 0.25-0.81). The acceptance of sharing health data for research was high (56%), and privacy concerns did not significantly affect routine engagement (OR: 0.81; 95% CI: 0.40-1.77). Results were consistent across race and gender.

**Conclusions:**

mHealth interventions have the potential to be scaled across populations. Optimizing applications to improve self-monitoring and personalization could increase routine engagement and, thus, user retention and intervention effectiveness.

Cardiovascular disease is the leading cause of death in North America, resulting in more than 750,000 deaths each year.^1^^,^^2^ Current data further suggest that the incidence of cardiovascular disease is increasing worldwide, with one-third of deaths globally being attributed to cardiovascular causes.^3^ Although population growth and aging explain a significant extent of these trends, the increasing prevalence of sedentary behaviors and attendant metabolic syndromes (eg, abdominal obesity, insulin resistance) are important contributable factors.^4^, ^5^, ^6^

As a result, primary cardiovascular prevention and physical activity promotion have become increasingly important in public health. Recently, mobile health (mHealth) technologies have expanded into this field given their greater potential reach and cost-effectiveness compared to traditional healthcare networks.^7^^,^^8^ Notably, mHealth technologies are currently being used for widespread primary prevention through health-promoting smartphone applications and studies that aim to increase daily physical activity while collecting data for research and program optimization.^9^ However, the effectiveness of mHealth interventions has generally been poor^10^ and is dependent on user attitudes and application engagement, which influence user retention, changes in behavior, and ultimately program effectiveness.^11^^,^^12^ Consequently, as the development of mHealth interventions and applications grows, researchers and developers need a better understanding of the motivators and predictors of engagement in mHealth applications, especially those that promote physical activity and fitness. The Unified Theory of Acceptance and Use of Technology has previously been shown to be associated with the intention to use mHealth applications.^13^^,^^14^ However, the influence of specific features of mHealth apps on use has not been widely studied. To address this knowledge gap and inform effective intervention design, we conducted a quantitative survey analysis to evaluate which specific features and barriers are most associated with the use and engagement of mHealth fitness applications. Furthermore, we assessed attitudes regarding data privacy within mHealth fitness applications and the acceptability of various privacy solutions.

## Methods

This cross-sectional study was described in accordance with the consensus-based checklist for reporting of survey studies.^15^ Adherence to the guidelines is demonstrated in Supplemental Table 1.

### Study development and study questionnaire

A mixed-methods approach was used to develop and assess the validity of the study questionnaire. Prior to evaluating attitudes and factors associated with mHealth application use and engagement, 10 semistructured cognitive interviews were performed to guide the development of the study questionnaire (Supplemental Text 1). Subsequently, a panel of experts comprising of clinicians (including cardiologists and internists), decision science experts, and institutional review board members evaluated the questionnaire.

Following a quantitative and thematic analysis of responses from our target demographic group, we developed a questionnaire consisting of 57 questions eliciting demographic characteristics in addition to 6 thematic topics evaluating attitudes and experiences related to personal health and mHealth fitness applications. The 6 thematic topics encompassed general well-being, past efforts to improve physical fitness, barriers to improving physical fitness, past experience with mHealth fitness applications, attitudes toward data privacy and research, and attitudes toward associated costs and benefits. The majority of questions were scored on a 5-point Likert scale with responses ranging from “strongly disagree” to “strongly agree.” The complete questionnaire, including the potential predictors of application use and engagement, is available in Supplemental Text 2.

### Endpoints of interest

The co-primary endpoints for this study were the use and routine use of an mHealth fitness application, which were, respectively, evaluated using binary and multiple-choice responses. Routine use of an mHealth application was used as a proxy for application engagement, and it was indicated when members of the target population reported using an mHealth fitness application on a “daily” or “weekly” basis.

### Study population and participant recruitment

Following ethics approval at the McGill University Health Centre, adult participants were recruited through single-stage random sampling. That is, individuals were recruited to participate in our mHealth application survey between September 15 and September 21, 2022, using the Prolific platform, which consists of a database of over 130,000 people registered to participate in crowdsourced behavioral research.^16^ Eligibility criteria included currently living in Canada, an age of 18 years or older, owning an Android or iOS smartphone, and having regular access to the internet via a smartphone data plan or high-speed broadband connection.

An emphasis was placed on recruiting participants from demographic groups frequently underrepresented in research including women, individuals from rural communities, and the elderly. This was done by launching additional demographic-specific studies on the Prolific platform. Participants who consented to participate in the survey were directed to an anonymous online questionnaire hosted on Surveymonkey.com.^17^ The estimated time to complete the survey was 10 minutes. Participants were provided a maximum of 44 minutes to complete the survey, at which point they were flagged for inactivity and removed from the study. Compensation for the completion of the survey was $2.14 Canadian Dollars per participant. Multiple participation was prevented on the Prolific platform and SurveyMonkey.com by reviewing participants’ Internet Protocol addresses.

### Statistical analysis

Results of the survey were described using proportions in percentage, mean ± SD deviations, or median and interquartile range (IQR), as appropriate. In addition, univariate logistic regression was performed to identify participant characteristics and mHealth application features that were facilitators or barriers to the co-primary outcomes. All survey items were checked for missingness and transformed into dichotomous variables. To dichotomize 5-point Likert scale responses (eg, [5] “strongly disagree”; [4] “disagree”; [3] “neither agree nor disagree”; [2] “agree”; [1] “strongly agree”), a logical condition coding the 3 bottom levels (5-3) as null and the 2 top levels (2-1) as positive was used.

The binary logistic models were not adjusted as the multicollinearity assumption was not met for multivariable regression.^18^ ORs and 95% CI were provided to quantify the association between the co-primary outcomes and the survey response items. Further, global *P* values corrected for multiple hypothesis testing with the Benjamini-Hochberg procedure were computed assuming a false discovery rate of 5%.^19^ In addition, subgroup analyses stratified according to race and gender were conducted to inform equity, diversity, and inclusion-based design. In some instances, the sample size was insufficient in the subgroup analyses for the logit models to generate reliable estimates, leading to the presence of NA values in the supplemental results. Descriptive variables were described with counts (proportions) and median (IQR) as appropriate. All analyses were performed with R Statistical Software (v4.1.3, R Core Team 2022).

## Results

### Participant characteristics

A total of 694 people completed the quantitative survey (93.3% completion rate) (Table 1). Among them, 48% (N = 333) reported using an mHealth fitness application, with 96% of users finding the apps convenient. Popular apps included Apple Health, Google Fit, Garmin Connect, Strava, and Garmin Connect. The use of mHealth applications was approximately equal in men (46.4%) and women (50%), as were most other demographic characteristics (Supplemental Table 2). The prevalence of current mHealth fitness application use was approximately equal in both men (46.4%) and women (50%), as were most other demographic characteristics (Supplemental Table 2). The majority of the cohort were female (62%), White Caucasian (67%), and Asian (26%), and the median age was 28 years (IQR 23-39 years, range 18-78 years). Most respondents were from urban (61%) and suburban (33%) areas and had at least a high school diploma (56%). Missing data were minimal (<%5%).

**Table 1:** Socioeconomic and Demographic Characteristics of Respondents

Gender	
Woman	427 (62.0)
Man	250 (36.0)
Nonbinary	14 (2.0)
Abstain	3 (0.4)
Age, y	
18-25	266 (38.5)
26-35	209 (30.2)
36-45	82 (11.9)
46-55	39 (5.6)
56+	95 (13.7)
Missing	3 (0.4)
Ethnicity	
White	468 (67.4)
Asian or Pacific Islander	177 (25.5)
Black or African American	29 (4.2)
Latinx	15 (2.2)
Indigenous	5 (0.7)
Geographic area	
Urban	422 (60.8)
Suburban	229 (33.0)
Rural	43 (6.2)
Marital status	
Married	184 (26.5)
Single	376 (54.2)
Common law	100 (14.4)
Separated	30 (1.3)
Widowed	4 (0.6)
Employment	
Full-time employment	320 (46.1)
Part-time employment	161 (23.2)
Unemployed	116 (16.7)
Not in labor force	98 (14.1)
Income	
Under $49,999	281 (40.5)
$50,000-$100,000	227 (32.7)
$100,000 or more	186 (26.8)
Education	
Elementary degree or less	7 (1.0)
High school diploma	109 (15.7)
Some college	138 (19.9)
Bachelor’s degree	277 (39.9)
Masters’ degree	70 (10.1)
Professional degree	17 (2.4)
Associate degree	63 (9.1)
Doctoral degree	13 (1.9)

Values are n (%).

### Personal health and activity goals

**Figure 1:**
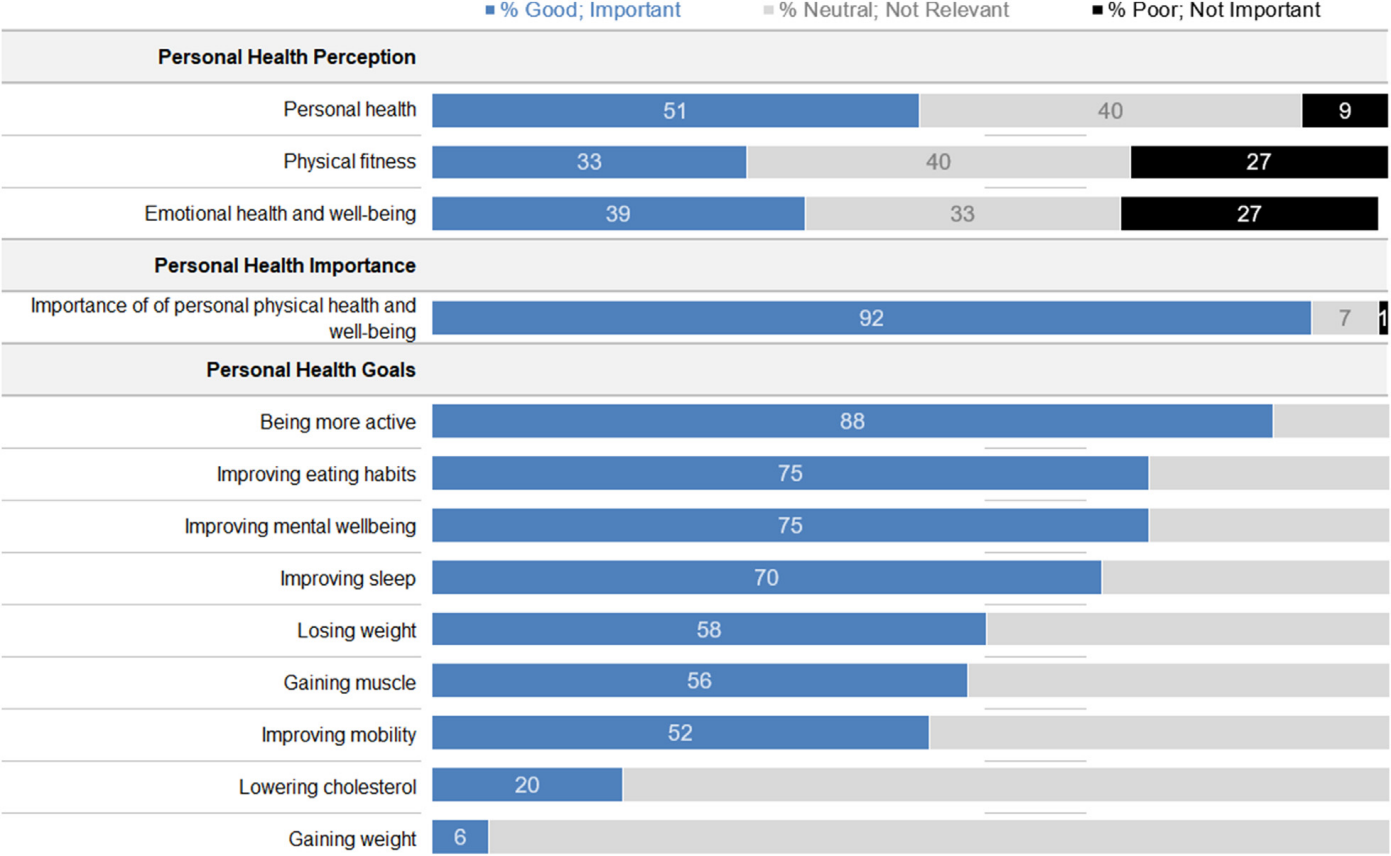
Survey Respondent’s Motivations and Perceptions of Personal Health

Most participants had a positive attitude toward their health. When asked about their personal health, personal fitness, and emotional well-being, 51%, 33%, and 39% of people, respectively, rated these aspects of themselves as positive (Figure 1). When asked how important their personal health and well-being were, 639 (92%) respondents reported that it was important or very important. When these people were asked to assess the most important aspects of their health, the most frequent responses were becoming more active (88%), improving eating habits (75%), and improving mental well-being (75%), while the least frequent responses were reducing cholesterol (20%) and gaining weight (eg, muscle; 6%).

### Factors associated with current use of mHealth applications

Within the overall cohort (n = 694), 3 factors were significantly associated with the current usage of an mHealth fitness application (n = 333). These were perceived importance of physical fitness (OR: 1.52; 95% CI: 1.11-2.10), the perceived importance of personal health and well-being (OR: 2.40; 95% CI: 1.34-4.50), and the interest to lose weight (OR: 2.11; 95% CI: 1.55-2.87) (Supplemental Table 3). There were several gender differences (Supplemental Table 4): the importance of mental health and well-being was more significantly associated with application use among participants who identified as Asian or Pacific Islanders (OR: 3.21; 95% CI: 1.72, 6.11; *P* interaction = 0.01), and the desire to improve sleep was more significantly associated with application use among men (OR: 1.76; 95% CI: 1.04-3.02; *P* interaction = 0.02).

### Facilitators of routine engagement

Table 2 describes attitudes and features associated with engagement in mHealth fitness applications among the cohort of current mHealth fitness application users (n = 333). Findings are summarized in the Central Illustration. Goal-setting and self-monitoring features were among the most important. People who felt mHealth fitness application helped them track progress toward their goals (OR: 5.10; 95% CI: 2.73-9.61), find and maintain motivation (OR: 4.11; 95% CI: 2.40-7.07), or maintain accountability (OR: 3.64; 95% CI: 2.13-6.23) were significantly more likely to use mHealth fitness applications on a regular basis. In addition, people who tracked daily steps taken (OR: 2.42; 95% CI: 1.44-4.07), heart rate (OR: 3.51, 95% CI: 1.94-6.69), calories or macronutrients (OR: 2.32; 95% CI: 1.38-4.00), or sleep (OR: 3.65; 95% CI: 1.86-7.85) were significantly more likely to engage with an mHealth fitness application. Other features associated with engagement included features allowing people to set (OR: 2.18; 95% CI: 1.29-3.75) and visualize (OR: 2.62; 95% CI: 1.56-4.46) concrete goals. No interactions were observed in subgroup analyses (all *P* interaction > 0.05) (Supplemental Table 5).

**Table 2:** Factors Associated With Routine Daily or Weekly Physical Activity Application Use

	OR (95% CI)	*P* Value	Q Value^a^
Perceived utility and convenience			
Apps help track progress toward my goals		**<0.001**	**<0.001**
Disagree	—		
Agree	5.10 (2.73-9.61)		
Apps help me find and maintain motivation		**<0.001**	**<0.001**
Disagree	—		
Agree	4.11 (2.40-7.07)		
Apps help keep me accountable toward my goals		**<0.001**	**<0.001**
Disagree	—		
Agree	3.64 (2.13-6.23)		
Apps are convenient to use		0.10	0.20
Disagree	—		
Agree	2.57 (0.82-7.64)		
Goals tracked			
Tracking daily steps taken		**<0.001**	**0.005**
No	—		
Yes	2.42 (1.44-4.07)		
Tracking time spent exercising		**0.034**	0.093
No	—		
Yes	1.74 (1.04-2.91)		
Tracking heart rate		**<0.001**	**<0.001**
No	—		
Yes	3.51 (1.94-6.69)		
Tracking calories or macronutrients		**0.001**	**0.009**
No	—		
Yes	2.32 (1.38-4.00)		
Tracking sleep		**<0.001**	**<0.001**
No	—		
Yes	3.65 (1.86-7.85)		
Positively perceived applications features			
Push notifications with exercise reminders		0.30	0.50
Disagree	—		
Agree	1.32 (0.76-2.34)		
Features allowing concrete goal setting		**0.003**	**0.014**
Disagree	—		
Agree	2.18 (1.29-3.75)		
Ability to visualize progress toward goals		**<0.001**	**0.002**
Disagree	—		
Agree	2.62 (1.56-4.46)		
Short motivational messages delivered through text		>0.90	>0.90
Disagree	—		
Agree	1.05 (0.46-2.74)		

Statistical significance is indicated by bold values when the significance level is below 0.05.

a False discovery rate correction for multiple testing.

**Central Illustration:**
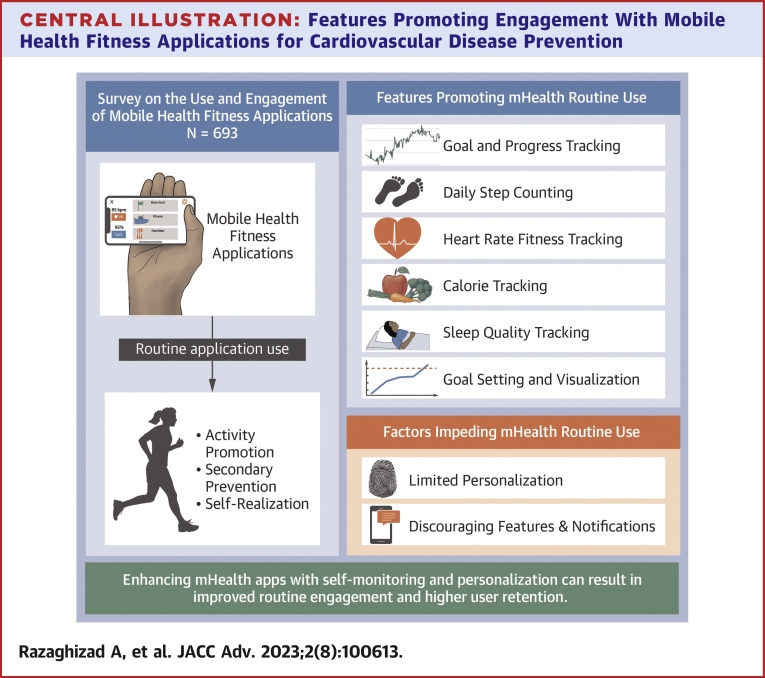
Features Promoting Engagement With Mobile Health Fitness Applications for Cardiovascular Disease Prevention

### Barriers to routine engagement

Barriers to engagement with mHealth fitness applications among current mHealth fitness application users (n = 333) are described in Table 3. The most prohibiting factors were those related to participants’ attitudes. People who did not believe they were motivated enough to be physically active (OR: 0.43; 95% CI: 0.25-0.73) or who believed they did not have the self-control to follow through on their attempts to be more active (OR: 0.46; 95% CI: 0.27-0.76) were about 2 times less likely to use an application on a routine basis. Applications that had limited personalization for goal setting (OR: 0.44; 95% CI: 0.25-0.81) or that were discouraging when goals were not met (OR: 0.48; 95% CI: 0.29-0.81) also demonstrated a significant negative association with engagement. Minor racial differences were observed (Supplemental Table 6); limited access to recreational facilities had the most impact on White individuals (OR: 0.47; 95% CI: 0.22-1.02; *P* interaction = 0.04), while the importance of evidence-based strategies was most important for people who identified as Asian or Pacific Islanders (OR: 0.36; 95% CI: 0.10-1.24; *P* interaction = 0.03), suggesting cultural differences in mHealth application use.

**Table 3:** Barriers Associated With Routine Daily or Weekly Physical Activity Application Use

	OR (95% CI)	*P* Value	Q Value^a^
Lifestyle barriers to physical activity			
Not enough time to being more physically active		0.50	0.70
Disagree	—		
Agree	1.20 (0.71-2.09)		
Not motivated enough to be more physically active		**0.002**	**0.009**
Disagree	—		
Agree	0.43 (0.25-0.73)		
Limited self-control to follow through on efforts		**0.003**	**0.013**
Disagree	—		
Agree	0.46 (0.27-0.76)		
Limited access to recreational facilities		0.060	0.15
Disagree	—		
Agree	0.57 (0.32-1.02)		
Limited energy or rest in day-to-day life		**0.020**	0.064
Disagree	—		
Agree	0.54 (0.32-0.91)		
Professional life too demanding		0.10	0.20
Disagree	—		
Agree	1.55 (0.92-2.66)		
Personal or home life too demanding		0.50	0.60
Disagree	—		
Agree	1.24 (0.69-2.29)		
Negatively perceived application features			
Too many notifications		0.10	0.20
Disagree	—		
Agree	0.65 (0.39-1.08)		
Discouraged when goals unmet		**0.006**	**0.022**
Disagree	—		
Agree	0.48 (0.29-0.81)		
Limited personalization for goal setting		**0.009**	**0.032**
Disagree	—		
Agree	0.44 (0.25-0.81)		
Application too expensive		>0.90	>0.90
Disagree	—		
Agree	1.03 (0.57-1.95)		
Limited perceived utility		0.073	0.20
Disagree	—		
Agree	0.56 (0.30-1.06)		
Insufficient number of features		**0.019**	0.063
Disagree	—		
Agree	0.54 (0.32-0.90)		
Application did not meet accessibility standards		0.20	0.40
Disagree	—		
Agree	0.45 (0.12-1.78)		
Application required expensive peripheral devices		0.12	0.20
Disagree	—		
Agree	0.59 (0.31-1.14)		
Application was difficult to use		0.30	0.50
Disagree	—		
Agree	2.49 (0.45-46.6)		
Limited esthetic appeal		>0.90	>0.90
Disagree	—		
Agree	0.97 (0.52-1.91)		
Application had data privacy concerns		0.60	0.70
Disagree	—		
Agree	0.81 (0.40-1.77)		
Application was not evidence based		0.70	0.80
Disagree	—		
Agree	0.83 (0.38-1.96)		

Statistical significance is indicated by bold values when the significance level is below 0.05.

a False discovery rate correction for multiple testing.

### Data privacy and data sharing

**Figure 2:**
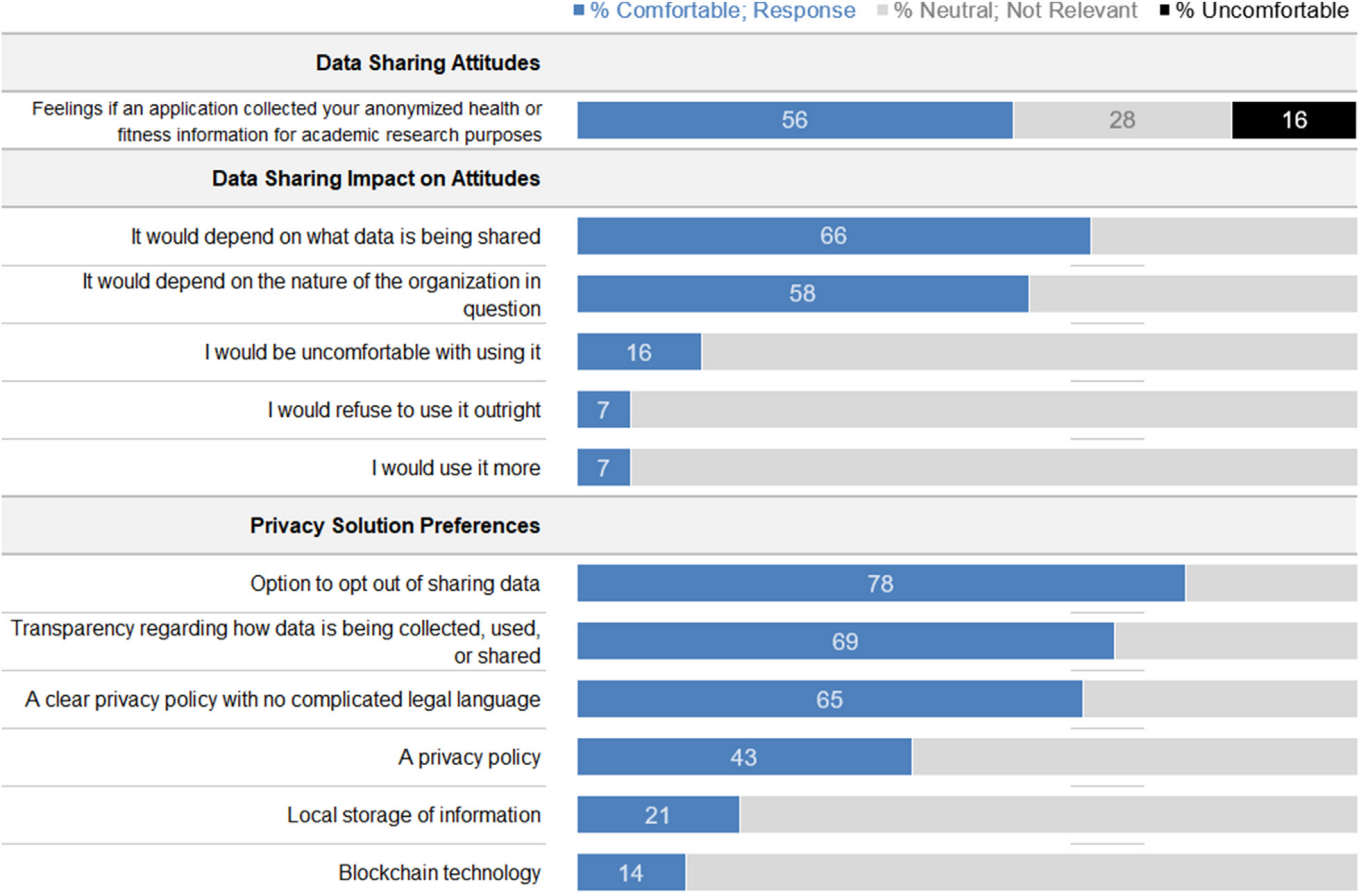
Survey Respondent’s Attitudes Regarding Physical Activity Data Sharing and Potential Solutions

The general attitude toward sharing personal anonymized health and fitness data for research purposes was positive among the overall cohort (n = 694); when asked to rate their opinions, 56% of respondents said they would be comfortable, 28% said they would be indifferent, and 16% said they would be uncomfortable (Figure 2). The impact of data sharing on participants’ attitudes was heterogeneous. When participants were asked how data sharing would affect their attitudes toward an mHealth fitness application, the most frequent responses were that it would depend on the data shared (66%) and the organization with whom the data would be shared (56%). When presented with various privacy solutions, the opportunity to opt out of sharing data (78%), transparency about how the data would be used (69%), and a clear privacy policy (65%) were the most acceptable solutions. Local storage of personal health data (21%) and blockchain encryption (14%) were less relevant privacy options.

## Discussion

This study was designed to evaluate mHealth features that were facilitators or barriers to the use and routine use of mHealth fitness applications, in addition to attitudes about data sharing for research purposes. The findings indicate that the perceived importance of physical health and well-being and the interest to lose weight are the factors most associated with mHealth application use. Moreover, self-monitoring and the ability to customize and visualize goals in mHealth fitness applications were the features most associated with engagement and routine use. Significant barriers to engagement consistently included the inability to set custom goals and application designs that were discouraging. The data also indicated that most individuals are comfortable with the idea of sharing their health and fitness data for research purposes.

To the best of our knowledge, this is the first quantitative analysis of features and barriers associated with the use and routine use of mHealth fitness applications. Our findings are consistent with a recent meta-analysis and meta-review that evaluated whether smartphone applications and activity monitors increase physical activity in adults. In the meta-analysis of 28 studies (n = 7,454), Laranjo and colleagues demonstrated that smartphone applications with personalization features were significantly more effective in meta-regression.^12^ This is consistent with our finding, which demonstrated that users were nearly half as likely to engage with mHealth fitness applications with limited goal customization.

These findings related to personalization have 2 significant implications for mHealth application and intervention design. The first is that a one-size-fits-all strategy for mHealth delivery and program design is unlikely to fulfill the needs of people who may benefit most from mHealth applications since personalization is a necessary component for behavioral change. This latter element is particularly significant since people differ in terms of personal preferences, activity levels, personal health goals, lifestyles, health problems, and personalities.^20^ The second implication is that researchers planning or developing innovative mHealth fitness applications should incorporate personalization features in order to increase application engagement and, thus, user retention and intervention efficacy.^11^

The use of mHealth fitness applications is also increasing in medicine and public health as a means to collect data and conduct research. This expansion is due to the multiple benefits that mHealth applications provide over traditional academic healthcare networks. These benefits include the flexibility to initiate interventions and capture data at any time or location, the ability to overcome obstacles to face-to-face interaction, and the ability to reduce inequities in accessibility by leveraging the ubiquity of smartphone devices.^21^ In North America and Europe, publicly available applications such as MyHeart Counts, MyFitness Counts, All of Us, and Cloudy with a Chance of Pain have aimed to better understand health conditions through routine data collection and parallel health promotion.^22^, ^23^, ^24^, ^25^ Our findings have shown that attitudes toward data sharing within mHealth applications for research purposes are generally positive in these populations, indicating potential for expansion in this space. However, considering issues around data security, our findings serve to highlight that future research-focused mHealth applications should include options to opt-in or opt-out of data sharing and transparency regarding the use of participant data (eg, what data will be used, how it will be used, and who will use it) to increase user confidence and participation in research programs.

As the use of mHealth applications increases as a means to conduct research and improve public health, user retention in mHealth applications becomes progressively more important. In terms of producing behavioral change, interventions are only effective to the extent that users adhere to and are retained within prescribed programs.^12^ With respect to research, disengagement can have significant effects on data integrity and the internal and external validity of mHealth studies.^26^ That is, if mechanisms of disengagement are not completely random (ie, if people who disengage are systematically different from those who do not), significant bias can be introduced by the means of missing data.^27^ Given that 71% of app users across all industries disengage from smartphone applications within 90 days,^28^ our findings emphasize the importance of incorporating effective app personalization and visual goal-setting and self-monitoring features, which we have shown to be significantly associated with routine application use. Notably, these findings are consistent with Susan Michie and colleagues’ behavioral change theory, which recognizes reflective processes (ie, evaluations and plans) as a central tenet for behavioral modification. Although routine engagement can be dependent on the time of application adoption,^26^ empirical evidence has shown routine engagement is associated with long-term user retention, which in turn is significantly associated with mHealth effectiveness.^11^^,^^12^

### Future directions and accessibility

There are several avenues for future research. First, studies, including the meta-regression by Laranjo et al, have demonstrated an association between mHealth application text messages and their overall effectiveness.^12^^,^^29^ This is inconsistent with our finding that the perceived utility of push notifications was not associated with routine use of mHealth fitness applications. Future experimental studies could aim to elucidate the cause of this discrepancy. Second, this study was not able to assess the impact of networking effects or community engagement on mHealth application engagement. As a result, future studies are needed to evaluate the impact and utility of such features, which enable users to improve connectivity within their communities.

Finally, although the absence of accessibility features was not significantly associated with engagement in our study, it did demonstrate a significant trend toward reduced application use (OR: 0.45; 95% CI: 0.12-1.78). As a result, as more researchers aim to design health interventions inside mHealth apps, accessibility features such as specific language options, compatibility with mobile operating systems' ability to deliver text-to-speech, and customizable font sizes within apps need to be prioritized to increase the reach and effectiveness of mHealth applications across populations. Furthermore, future research should leverage more participatory approaches that can draw out insights from groups most likely to benefit from mHealth tools. In particular, the involvement of user groups and feedback collection during application development has been shown to increase the frequency of use of mHealth applications and thus their potential impact.^30^

### Study Limitations

This study had several limitations. First, this was a single-country survey, and the majority of participants had high technological proficiency, came from higher educational backgrounds, and had a younger median age compared to the North American average.^31^ Consequently, only 9% of individuals rated their health as poor, which limits the generalizability of our findings to sicker individuals who may benefit most from mHealth-facilitated secondary prevention. Nevertheless, physical activity and its benefits are independent of demographic characteristics.^32^ Second, this study was conducted in a relatively general population of healthy and computer-literate adults. This means that the personal health perceptions and perceived utility of mHealth applications in people with acute and chronic conditions were not captured. Finally, the role of other extrinsic features that can influence physical activity, such as environment and weather, are challenging to address and identify irrespective of the ability to be motivated through a mobile application.

## Conclusions

Physical activity promotion through mHealth application has a significant potential to scale rapidly across a population. Prior research has shown that user- and human-centered approaches to the design and development of mHealth applications are major predictors of their success.^33^ To that end, we conducted a quantitative analysis of factors that facilitate use and engagement with mHealth applications to optimize their delivery and design. Our findings demonstrate that the motivation to reduce body weight is significantly associated with the usage of mHealth fitness applications. Furthermore, our data show goal visualization, goal customization, and self-monitoring (eg, daily steps, heart rate, calories, or sleep) are significantly associated with routine use and engagement with mHealth fitness applications. The consideration of such aspects in the design and development of health-promoting mHealth applications may result in increased engagement, application retention, and program effectiveness.

PERSPECTIVES **COMPETENCY IN SYSTEMS-BASED PRACTICE:** Emphasizing personal health importance can promote mHealth engagement. Incorporating goal tracking features enhances routine use of mHealth applications, improving intervention effectiveness.

**TRANSLATIONAL OUTLOOK:** Customization of goals within mHealth apps is crucial to drive user engagement. Exploring secure and effective methods for sharing health data can facilitate large-scale research. Addressing barriers and optimizing mHealth implementation will enhance translation into clinical practice.

## Funding support and author disclosures

This study was funded by LEAP-Pecaut Centre, 10.13039/100011094Public Health Agency of Canada, 10.13039/100014131McGill University Health Centre (MUHC) Foundation, 10.13039/501100017657Montreal General Hospital (MGH) Foundation, Sarah Louise King Award, Marjorie Cadham Award, Inez and Willena Beaton Award, Canso foundation, Department of Medicine at McGill University Health Centre, and Fonds de Recherche Santé Quebec (FRSQ) Junior 1 clinician scholars’ program. The Canadian Institute of Health Research (Grant # 175095), Fonds de Recherche Santé Quebec (FRSQ) Junior 1 clinician scholars' program, Alberta Innovates Health Solution, European Society of Cardiology young investigator grant, Roche Diagnostics, Boeringer-Ingelheim, Novartis, and Takeda support Dr Sharma. Dr Friedrich is co-founder of AiVALON, a Montreal-based company that produces mobile health software. The authors have reported that they have no relationships relevant to the contents of this paper to disclose.
