# Supplementary material for: Mobile Health Fitness Interventions: Impact of Features on Routine Use and Data Sharing Acceptability
Source: JACC Adv. 2023 Sep 22;2(8):100613. doi: 10.1016/j.jacadv.2023.100613 (PMC11198255; doi:10.1016/j.jacadv.2023.100613)
Supplement: Supplementary data [file mmc1.pdf]

## SUPPLEMENTAL APPENDIX

Supplemental Text 1. Questioned Administered in Semi-Structured Cognitive Interviews

1. How would you characterize your current personal health and well-being?
2. What, if anything, would you like to improve about your health and well-being?
3. When considering all the things you do on a daily basis, how important is getting physical activity?
4. Why do you believe it is important or unimportant?
5. Are you interested in increasing your daily levels of physical activity?
6. What barriers do you face when trying to improve your physical activity?
7. What has worked for you in past attempts to improve your own physical activity habits?
8. Do you currently use any health and fitness apps on your phone?
9. Which health and fitness apps do you use?
10. Could you describe the role these apps play in your life?
11. What are your favorite features?
12. How frequently do you prefer to use them?
13. Is there anything you would change about these applications?
14. Have you tried using any health and fitness apps in the past? If so, why did you stop using them?
15. Why do you not feel the need to use health and fitness apps?
16. Do you have any desire to try applications focused on health and fitness? If so, what would motivate you to try one in particular?
17. What motivates you to use or consider using mobile applications that target health and wellness?
18. What prevents you to use or consider using mobile applications that target health and wellness?
19. Have you experienced any significant cultural or social barriers to improving your health and wellness (e.g., my friends or family don't value it, so I don't either)?
20. Have you experienced any significant physical barriers to improving your health and wellness (e.g., areas where I can exercise are inaccessible or expensive)?
21. Do you evaluate the credibility and quality of an app before you download it? If so, how?
22. Do you have any concerns about the privacy and security of your personal information when using a health or fitness app?
23. What security features would give you more confidence in using the app?
24. Would you be comfortable if a wellness application collected your anonymized health or fitness information explicitly for academic research purposes, and with your consent? For clarity, "anonymized" entails the removal of any personal identifiable information like name, email address, birthday, etc.

Supplemental Text 2. Administered Survey (see the end of this document)

Supplemental Table 1. Consensus-Based Checklist for Reporting of Survey Studies (CROSS) Guidelines

| Section/topic | Item | Item description | Reported on page # |
| --- | --- | --- | --- |
| **Title and abstract** | | | |
| Title and abstract | 1a | State the word "survey" along with a commonly used term in title or abstract to introduce the study's design. | 2 |
| | 1b | Provide an informative summary in the abstract, covering background, objectives, methods, findings/results, interpretation/discussion, and conclusions. | 2 |
| **Introduction** | | | |
| Background | 2 | Provide a background about the rationale of study, what has been previously done, and why this survey is needed. | 3 |
| Purpose/aim | 3 | Identify specific purposes, aims, goals, or objectives of the study. | 3 |
| **Methods** | | | |
| Study design | 4 | Specify the study design in the methods section with a commonly used term (e.g., cross-sectional or longitudinal). | 4 |
| | 5a | Describe the questionnaire (e.g., number of sections, number of questions, number and names of instruments used). | 4 |
| Data collection methods | 5b | Describe all questionnaire instruments that were used in the survey to measure particular concepts. Report target population, reported validity and reliability information, scoring/classification procedure, and reference links (if any). | 4 |
| | 5c | Provide information on pretesting of the questionnaire, if performed (in the article or in an online supplement). Report the method of pretesting, number of times questionnaire was pre-tested, number and demographics of participants used for pretesting, and the level of similarity of demographics between pre-testing participants and sample population. | NA |
| | 5d | Questionnaire if possible, should be fully provided (in the article, or as appendices or as an online supplement). | Text S1 |
| Sample characteristics | 6a | Describe the study population (i.e., background, locations, eligibility criteria for participant inclusion in survey, exclusion criteria). | 4 |
| | 6b | Describe the sampling techniques used (e.g., single stage or multistage sampling, simple random sampling, stratified sampling, cluster sampling, convenience sampling). Specify the locations of sample participants whenever clustered sampling was applied. | 4 |
| | 6c | Provide information on sample size, along with details of sample size calculation. | NA |
| | 6d | Describe how representative the sample is of the study population (or target population if possible), particularly for population-based surveys. | 4 |
| Survey administration | 7a | Provide information on modes of questionnaire administration, including the type and number of contacts, the location where the survey was conducted (e.g., outpatient room or by use of online tools, such as SurveyMonkey). | 4 |
| | 7b | Provide information of survey's time frame, such as periods of recruitment, exposure, and follow-up days. | 4 |

| | | | |
|---|---|---|---|
| | 7c | Provide information on the entry process:<br>• For non-web-based surveys, provide approaches to minimize human error in data entry.<br>• For web-based surveys, provide approaches to prevent “multiple participation” of participants. | 4 |
| Study preparation | 8 | Describe any preparation process before conducting the survey (e.g., interviewers’ training process, advertising the survey). | NA |
| Ethical considerations | 9a | Provide information on ethical approval for the survey if obtained, including informed consent, institutional review board [IRB] approval, Helsinki declaration, and good clinical practice [GCP] declaration (as appropriate). | 4 |
| | 9b | Provide information about survey anonymity and confidentiality and describe what mechanisms were used to protect unauthorized access. | 4 |
| Statistical analysis | 10a | Describe statistical methods and analytical approach. Report the statistical software that was used for data analysis. | 5 |
| | 10b | Report any modification of variables used in the analysis, along with reference (if available). | 5 |
| | 10c | Report details about how missing data was handled. Include rate of missing items, missing data mechanism (i.e., missing completely at random [MCAR], missing at random [MAR] or missing not at random [MNAR]) and methods used to deal with missing data (e.g., multiple imputation). | 5 |
| | 10d | State how non-response error was addressed. | NA |
| | 10e | For longitudinal surveys, state how loss to follow-up was addressed. | NA |
| | 10f | Indicate whether any methods such as weighting of items or propensity scores have been used to adjust for non-representativeness of the sample. | NA |
| | 10g | Describe any sensitivity analysis conducted. | 5 |
| **Results** | | | |
| Respondent characteristics | 11a | Report numbers of individuals at each stage of the study. Consider using a flow diagram, if possible. | 6 |
| | 11b | Provide reasons for non-participation at each stage, if possible. | NA |
| | 11c | Report response rate, present the definition of response rate or the formula used to calculate response rate. | NA |
| | 11d | Provide information to define how unique visitors are determined. Report number of unique visitors along with relevant proportions (e.g., view proportion, participation proportion, completion proportion). | NA |
| Descriptive results | 12 | Provide characteristics of study participants, as well as information on potential confounders and assessed outcomes. | 6 |
| Main findings | 13a | Give unadjusted estimates and, if applicable, confounder-adjusted estimates along with 95% confidence intervals and p-values. | Tables |

| | | | |
|---|---|---|---|
| | 13b | For multivariable analysis, provide information on the model building process, model fit statistics, and model assumptions (as appropriate). | NA |
| | 13c | Provide details about any sensitivity analysis performed. If there are considerable amount of missing data, report sensitivity analyses comparing the results of complete cases with that of the imputed dataset (if possible). | 6 |
| **Discussion** | | | |
| Limitations | 14 | Discuss the limitations of the study, considering sources of potential biases and imprecisions, such as non-representativeness of sample, study design, important uncontrolled confounders. | 11 |
| Interpretations | 15 | Give a cautious overall interpretation of results, based on potential biases and imprecisions and suggest areas for future research. | 8–12 |
| Generalizability | 16 | Discuss the external validity of the results. | 11 |
| **Other sections** | | | |
| Role of funding source | 17 | State whether any funding organization has had any roles in the survey's design, implementation, and analysis. | 12 |
| Conflict of interest | 18 | Declare any potential conflict of interest. | 12 |
| Acknowledgements | 19 | Provide names of organizations/persons that are acknowledged along with their contribution to the research. | 12 |

Abbreviations: NA = Not Applicable

Supplemental Table 2.

| Variable | Users of health or fitness apps N = 333 | Percent | Non-users of health or fitness apps N = 361 | Percent |
|---|---|---|---|---|
| Gender | | | | |
| Woman | 212 | 64% | 215 | 60% |
| Man | 116 | 35% | 134 | 37% |
| Non-Binary | 4 | 1% | 10 | 3% |
| Abstain | 1 | 0% | 2 | 1% |
| Age | | | | |
| 18-25 | 124 | 37% | 142 | 40% |
| 26-35 | 111 | 33% | 98 | 27% |
| 36-45 | 38 | 11% | 44 | 12% |
| 46-55 | 17 | 5% | 22 | 6% |
| 56+ | 42 | 13% | 53 | 15% |
| Ethnicity | | | | |
| White | 227 | 65% | 241 | 64% |
| Hispanic or Latino | 11 | 3% | 9 | 2% |
| Black or African American | 16 | 5% | 19 | 5% |
| First nations, Metis, or Inuit | 4 | 1% | 8 | 2% |
| Asian or Pacific Islander | 89 | 26% | 99 | 26% |
| Education | | | | |
| None | 0 | 0% | 1 | 0% |
| High school diploma | 39 | 12% | 70 | 19% |
| Some college, no degree | 59 | 18% | 79 | 22% |
| Associate degree | 0 | 0% | 0 | 0% |
| Bachelor's degree | 148 | 44% | 129 | 36% |
| Master's degree | 36 | 11% | 34 | 9% |
| Professional degree | 0 | 0% | 0 | 0% |
| Doctoral degree | 7 | 2% | 6 | 2% |
| Area | | | | |
| City/Urban | 210 | 63% | 212 | 59% |
| Rural | 15 | 5% | 28 | 8% |
| Suburban | 108 | 32% | 121 | 34% |
| Employment | | | | |
| Full-time employment | 178 | 53% | 142 | 39% |
| Part-time employment | 69 | 21% | 92 | 25% |
| Unemployed, job searcher/seeker | 39 | 12% | 46 | 13% |
| Unemployed, left job | 8 | 2% | 10 | 3% |
| Unemployed, temporary layoff | 2 | 1% | 3 | 1% |
| Unemployed, lost job | 3 | 1% | 4 | 1% |
| Not in labor force | 34 | 10% | 64 | 18% |
| Income | | | | |
| Under $49,999 | 128 | 38% | 150 | 42% |
| $50,000 to $74,999 | 109 | 33% | 118 | 33% |

| | | | | |
|---|---|---|---|---|
| $100,000 or more | 96 | 29% | 90 | 25% |

Supplemental Table 3. Factors Associated with mHealth Fitness Application Use

| Characteristic | OR[1] | 95% CI[1] | p-value | q-value[2] |
|---|---|---|---|---|
| Perceptions of Person Health | | | | |
| Personal health | | | 0.4 | 0.6 |
| Poor | — | — | | |
| Good | 1.13 | 0.84, 1.53 | | |
| **Physical fitness** | | | **0.009** | **0.050** |
| Poor | — | — | | |
| Good | 1.52 | 1.11, 2.10 | | |
| Emotional health and well-being | | | 0.2 | 0.4 |
| Poor | — | — | | |
| Good | 1.23 | 0.91, 1.67 | | |
| **Importance of physical health and well-being** | | | **0.003** | **0.027** |
| Poor | — | — | | |
| Good | 2.40 | 1.34, 4.50 | | |
| Personal Wellness Goals | | | | |
| Interested in improving physical health and wellness | | | 0.2 | 0.4 |
| Disagree | — | — | | |
| Agree | 1.88 | 0.77, 5.01 | | |
| Interested in increasing daily physical activity | | | 0.8 | 0.9 |
| Disagree | — | — | | |
| Agree | 0.95 | 0.56, 1.60 | | |
| **Interested in losing weight** | | | **<0.001** | **<0.001** |
| Disagree | — | — | | |
| Agree | 2.11 | 1.55, 2.87 | | |
| Interested in being more active | | | 0.13 | 0.4 |
| Disagree | — | — | | |
| Agree | 1.48 | 1.01, 2.18 | | |
| Interested in increasing mobility | | | 0.5 | 0.6 |
| Disagree | — | — | | |
| Agree | 1.10 | 0.82, 1.49 | | |
| Interested in improving eating habits | | | **0.016** | 0.071 |
| Disagree | — | — | | |
| Agree | 1.50 | 1.08, 2.10 | | |
| Interested in lowering cholesterol | | | 0.2 | 0.4 |
| Disagree | — | — | | |
| Agree | 1.26 | 0.86, 1.84 | | |
| Interested in improving sleep | | | 0.3 | 0.6 |
| Disagree | — | — | | |
| Agree | 1.16 | 0.85, 1.60 | | |
| Interested in gaining muscle | | | 0.078 | 0.3 |
| Disagree | — | — | | |
| Agree | 1.31 | 0.97, 1.77 | | |
| Interested in improving mental well-being | | | 0.4 | 0.6 |
| Disagree | — | — | | |

| | | |
|---|---|---|
| Agree | 1.16 | 0.84, 1.61 |

[1]OR = Odds Ratio, CI = Confidence Interval

[2]False discovery rate correction for multiple testing

Supplemental Table 4. Perceptions and Goals Associated with mHealth Fitness Application Use by Race and Gender

| Characteristic | OR[1] | 95% CI[1] | p-value | p-interaction |
|---|---|---|---|---|
| Perceptions of Person Health | | | | |
| Personal health | | | | |
| White | 1.05 | 0.73, 1.51 | 0.8 | 0.75 |
| Asian or Pacific Islander | 1.25 | 0.69, 2.26 | 0.47 | 0.75 |
| Black or African American | 1.33 | 0.24, 7.40 | 0.74 | 0.75 |
| First nations, Metis, or Inuit | NA | NA, NA | 0.98 | 0.75 |
| Hispanic or Latino | 2.5 | 0.29, 21.4 | 0.4 | 0.75 |
| Man | 1.06 | 0.64, 1.75 | 0.82 | 0.43 |
| Non-Binary | NA | NA, NA | 0.97 | 0.43 |
| Woman | 1.16 | 0.79, 1.70 | 0.44 | 0.43 |
| Physical fitness | | | | |
| White | 1.66 | 1.13, 2.44 | 0.01 | 0.42 |
| Asian or Pacific Islander | 1.07 | 0.55, 2.07 | 0.85 | 0.42 |
| Black or African American | 2 | 0.46, 8.78 | 0.36 | 0.42 |
| First nations, Metis, or Inuit | NA | NA, NA | 0.97 | 0.42 |
| Hispanic or Latino | 0.86 | 0.04, 16.85 | 0.92 | 0.42 |
| Man | 1.07 | 0.64, 1.78 | 0.8 | 0.14 |
| Non-Binary | NA | NA, NA | 0.98 | 0.14 |
| Woman | 1.91 | 1.26, 2.91 | 0.003 | 0.14 |
| Emotional health and well-being | | | | |
| White | 0.94 | 0.65, 1.36 | 0.74 | **0.01** |
| Asian or Pacific Islander | 3.21 | 1.72, 6.11 | 0.0003 | **0.01** |
| Black or African American | 0.48 | 0.09, 2.27 | 0.36 | **0.01** |
| First nations, Metis, or Inuit | NA | NA, NA | 1 | **0.01** |
| Hispanic or Latino | 0.83 | 0.08, 9.2 | 0.88 | **0.01** |
| Man | 1.08 | 0.66, 1.78 | 0.76 | 0.51 |
| Non-Binary | NA | NA, NA | 0.98 | 0.51 |
| Woman | 1.36 | 0.92, 2.02 | 0.13 | 0.51 |
| Importance of physical health and well-being | | | | |
| White | 2.27 | 1.12, 4.92 | 0.028 | 0.66 |
| Asian or Pacific Islander | 2.17 | 0.77, 7.1 | 0.16 | 0.66 |
| Black or African American | NA | NA, NA | 1 | 0.66 |
| First nations, Metis, or Inuit | NA | NA, NA | 1 | 0.66 |
| Hispanic or Latino | NA | NA, NA | 1 | 0.66 |
| Man | 4.07 | 1.45, 14.47 | 0.01 | 0.08 |
| Non-Binary | NA | NA, NA | 0.98 | 0.08 |
| Woman | 2.08 | 1.00, 4.57 | 0.06 | 0.08 |
| Personal Wellness Goals | | | | |
| Interested in improving physical health and wellness | | | | |
| White | 1.42 | 0.40, 5.63 | 0.59 | 0.56 |
| Asian or Pacific Islander | 1.73 | 0.44, 8.43 | 0.45 | 0.56 |
| Black or African American | NA | NA, NA | 1 | 0.56 |
| First nations, Metis, or Inuit | NA | NA, NA | 1 | 0.56 |

| | | | | |
|---|---|---|---|---|
| Hispanic or Latino | NA | NA, NA | 1 | 0.56 |
| Man | 2.67 | 0.60, 18.48 | 0.23 | 0.61 |
| Non-Binary | NA | NA, NA | 1 | 0.61 |
| Woman | 1.6 | 0.52, 5.37 | 0.42 | 0.61 |
| Interested in increasing daily physical activity | | | | |
| White | 0.89 | 0.46, 1.69 | 0.72 | 0.95 |
| Asian or Pacific Islander | 1.08 | 0.38, 3.15 | 0.89 | 0.95 |
| Black or African American | 0.92 | 0.10, 8.71 | 0.94 | 0.95 |
| First nations, Metis, or Inuit | NA | NA, NA | 1 | 0.95 |
| Hispanic or Latino | NA | NA, NA | 1 | 0.95 |
| Man | 1.1 | 0.48, 2.59 | 0.82 | 0.67 |
| Non-Binary | NA | NA, NA | 1 | 0.67 |
| Woman | 0.87 | 0.44, 1.73 | 0.69 | 0.67 |
| Interested in losing weight | | | | |
| White | 1.84 | 1.27, 2.66 | 0.0014 | 0.62 |
| Asian or Pacific Islander | 2.54 | 1.38, 4.68 | 0.0029 | 0.62 |
| Black or African American | 3.24 | 0.69, 15.2 | 0.14 | 0.62 |
| First nations, Metis, or Inuit | 2 | 0.05, 78.25 | 0.71 | 0.62 |
| Hispanic or Latino | 9.33 | 0.71, 122.57 | 0.09 | 0.62 |
| Man | 2.74 | 1.64, 4.59 | 0.00013 | 0.46 |
| Non-Binary | 2 | 0.15, 26.73 | 0.6 | 0.46 |
| Woman | 1.81 | 1.23, 2.68 | 0.0028 | 0.46 |
| Interested in being more active | | | | |
| White | 1.6 | 0.97, 2.62 | 0.06 | 0.26 |
| Asian or Pacific Islander | 1.15 | 0.53, 2.49 | 0.73 | 0.26 |
| Black or African American | 0.3 | 0.05, 1.91 | 0.2 | 0.26 |
| First nations, Metis, or Inuit | NA | NA, NA | 0.98 | 0.26 |
| Hispanic or Latino | 1.2 | 0.12, 11.87 | 0.88 | 0.26 |
| Man | 1.88 | 0.98, 3.61 | 0.06 | 0.11 |
| Non-Binary | 0.11 | 0.006, 1.92 | 0.13 | 0.11 |
| Woman | 1.19 | 0.71, 1.99 | 0.5 | 0.11 |
| Interested in increasing mobility | | | | |
| White | 0.99 | 0.69, 1.42 | 0.94 | 0.2 |
| Asian or Pacific Islander | 1.67 | 0.91, 3.04 | 0.1 | 0.2 |
| Black or African American | 0.56 | 0.13, 2.51 | 0.45 | 0.2 |
| First nations, Metis, or Inuit | NA | NA, NA | 0.98 | 0.2 |
| Hispanic or Latino | 0.4 | 0.05, 3.42 | 0.4 | 0.2 |
| Man | 1.01 | 0.61, 1.67 | 0.96 | 0.23 |
| Non-Binary | 0.14 | 0.01, 1.99 | 0.15 | 0.23 |
| Woman | 1.2 | 0.82, 1.76 | 0.35 | 0.23 |
| Interested in improving eating habits | | | | |
| White | 1.6 | 1.07, 2.40 | 0.022 | 0.83 |
| Asian or Pacific Islander | 1.36 | 0.71, 2.66 | 0.36 | 0.83 |

| | | | | |
|---|---|---|---|---|
| Black or African American | 1.39 | 0.28, 7.23 | 0.69 | 0.83 |
| First nations, Metis, or Inuit | NA | NA, NA | 1 | 0.83 |
| Hispanic or Latino | 0.5 | 0.02, 6.68 | 0.61 | 0.83 |
| Man | 1.23 | 0.73, 2.10 | 0.43 | 0.09 |
| Non-Binary | 0.11 | 0.004, 1.70 | 0.13 | 0.09 |
| Woman | 1.84 | 1.20, 2.87 | 0.006 | 0.09 |
| Interested in lowering cholesterol | | | | |
| White | 1.09 | 0.68, 1.74 | 0.72 | 0.31 |
| Asian or Pacific Islander | 1.93 | 0.87, 4.30 | 0.11 | 0.31 |
| Black or African American | 0.42 | 0.06, 2.77 | 0.36 | 0.31 |
| First nations, Metis, or Inuit | 0.5 | 0.01, 19.56 | 0.71 | 0.31 |
| Hispanic or Latino | 6 | 0.48, 75.34 | 0.17 | 0.31 |
| Man | 1.17 | 0.65, 2.11 | 0.6 | 0.79 |
| Non-Binary | 0.78 | 0.06, 10.86 | 0.85 | 0.79 |
| Woman | 1.47 | 0.87, 2.50 | 0.15 | 0.79 |
| Interested in improving sleep | | | | |
| White | 1.35 | 0.92, 1.98 | 0.12 | 0.47 |
| Asian or Pacific Islander | 0.91 | 0.48, 1.72 | 0.76 | 0.47 |
| Black or African American | 0.68 | 0.11, 3.84 | 0.66 | 0.47 |
| First nations, Metis, or Inuit | NA | NA, NA | 1 | 0.47 |
| Hispanic or Latino | 0.4 | 0.039, 3.25 | 0.4 | 0.47 |
| Man | 1.76 | 1.04, 3.02 | 0.04 | **0.02** |
| Non-Binary | 0.08 | 0.003, 1.03 | 0.08 | **0.02** |
| Woman | 0.97 | 0.65, 1.44 | 0.86 | **0.02** |
| Interested in gaining muscle | | | | |
| White | 1.36 | 0.94, 1.96 | 0.1 | 0.12 |
| Asian or Pacific Islander | 1.51 | 0.83, 2.78 | 0.18 | 0.12 |
| Black or African American | 0.2 | 0.03, 1.0 | 0.061 | 0.12 |
| First nations, Metis, or Inuit | NA | NA, NA | 1 | 0.12 |
| Hispanic or Latino | 2.22 | 0.29, 19.84 | 0.45 | 0.12 |
| Man | 1.22 | 0.73, 2.04 | 0.44 | 0.32 |
| Non-Binary | 0.22 | 0.009, 2.48 | 0.26 | 0.32 |
| Woman | 1.42 | 0.97, 2.09 | 0.07 | 0.32 |
| Interested in improving mental well-being | | | | |
| White | 1.34 | 0.89, 2.00 | 0.16 | 0.27 |
| Asian or Pacific Islander | 0.96 | 0.51, 1.82 | 0.9 | 0.27 |
| Black or African American | 0.53 | 0.11, 2.56 | 0.43 | 0.27 |
| First nations, Metis, or Inuit | NA | NA, NA | 0.98 | 0.27 |
| Hispanic or Latino | 0.28 | 0.02, 3.58 | 0.33 | 0.27 |
| Man | 1.44 | 0.86, 2.42 | 0.17 | 0.43 |
| Non-Binary | 0.33 | 0.02, 7.14 | 0.48 | 0.43 |
| Woman | 1.01 | 0.61, 1.55 | 0.98 | 0.43 |

Supplemental Table 5. Factors Associated with Routine Daily or Weekly Physical Activity Application Use

| Characteristic | OR[1] | 95% CI[1] | p-value | p-interaction |
|---|---|---|---|---|
| Perceived Utility and Convenience | | | | |
| Apps help track progress towards my goals | | | | |
| White | 5.28 | 2.24, 12.44 | 0.0001 | 0.09 |
| Asian or Pacific Islander | 3.11 | 1.08, 8.97 | 0.04 | 0.09 |
| Black or African American | NA | NA, NA | 0.99 | 0.09 |
| First nations, Metis, or Inuit | NA | NA, NA | 1 | 0.09 |
| Hispanic or Latino | NA | NA, NA | 0.99 | 0.09 |
| Man | 3.33 | 1.08, 10.24 | 0.04 | 0.56 |
| Non-Binary | NA | NA, NA | 1 | 0.56 |
| Woman | 7.05 | 3.21, 15.49 | <0.0001 | 0.56 |
| Apps help me find and maintain motivation | | | | |
| White | 3.81 | 1.9, 7.63 | 0.0002 | 0.95 |
| Asian or Pacific Islander | 4.25 | 1.61, 11.21 | 0.003 | 0.95 |
| Black or African American | 11.0 | 0.35, 345.06 | 0.17 | 0.95 |
| First nations, Metis, or Inuit | NA | NA, NA | 1 | 0.95 |
| Hispanic or Latino | NA | NA, NA | 0.99 | 0.95 |
| Man | 2.92 | 1.17, 7.25 | 0.02 | 0.57 |
| Non-Binary | NA | NA, NA | 1 | 0.57 |
| Woman | 5.38 | 2.72, 10.64 | <0.0001 | 0.57 |
| Apps help keep me accountable towards my goals | | | | |
| White | 3.88 | 1.95, 7.73 | 0.0001 | 0.34 |
| Asian or Pacific Islander | 2.19 | 0.86, 5.54 | 0.1 | 0.34 |
| Black or African American | NA | NA, NA | 0.99 | 0.34 |
| First nations, Metis, or Inuit | NA | NA, NA | 1 | 0.34 |
| Hispanic or Latino | NA | NA, NA | 0.99 | 0.34 |
| Man | 2.72 | 1.1, 6.73 | 0.03 | 0.74 |
| Non-Binary | NA | NA, NA | 1 | 0.74 |
| Woman | 4.26 | 2.18, 8.33 | <0.0001 | 0.74 |
| Apps are convenient to use | | | | |
| White | 2.1 | 0.43, 8.29 | 0.31 | 0.83 |
| Asian or Pacific Islander | 2.72 | 0.43, 21.6 | 0.29 | 0.83 |
| Black or African American | NA | NA, NA | 1 | 0.83 |
| First nations, Metis, or Inuit | NA | NA, NA | 1 | 0.83 |
| Hispanic or Latino | NA | NA, NA | 1 | 0.83 |
| Man | 3.22 | 0.12, 83.3 | 0.41 | 0.86 |
| Non-Binary | NA | NA, NA | 1 | 0.86 |
| Woman | 2.46 | 0.70, 8.08 | 0.14 | 0.86 |
| Goals Tracked | | | | |
| Tracking daily steps taken | | | | |
| White | 1.99 | 1.03, 3.87 | 0.04 | 0.2 |
| Asian or Pacific Islander | 2.4 | 0.95, 6.03 | 0.06 | 0.2 |

| | | | | |
|---|---|---|---|---|
| Black or African American | NA | NA, NA | 0.99 | 0.2 |
| First nations, Metis, or Inuit | NA | NA, NA | 1 | 0.2 |
| Hispanic or Latino | NA | NA, NA | 0.99 | 0.2 |
| Man | 2.47 | 1.04, 5.97 | 0.04 | 0.91 |
| Non-Binary | NA | NA, NA | 1 | 0.91 |
| Woman | 2.63 | 1.98, 5.07 | 0.003 | 0.91 |
| Tracking time spent exercising | | | | |
| White | 1.88 | 0.97, 3.64 | 0.06 | 0.21 |
| Asian or Pacific Islander | 1.19 | 0.47, 2.96 | 0.72 | 0.21 |
| Black or African American | NA | NA, NA | 0.99 | 0.21 |
| First nations, Metis, or Inuit | NA | NA, NA | 1 | 0.21 |
| Hispanic or Latino | NA | NA, NA | 0.99 | 0.21 |
| Man | 1.44 | 0.61,3.42 | 0.4 | 0.49 |
| Non-Binary | NA | NA, NA | 1 | 0.49 |
| Woman | 2.11 | 1.11,4.03 | 0.023 | 0.49 |
| Tracking heart rate | | | | |
| White | 3.71 | 1.64, 8.41 | 0.002 | 0.56 |
| Asian or Pacific Islander | 2.49 | 0.91, 6.85 | 0.08 | 0.56 |
| Black or African American | NA | NA, NA | 0.98 | 0.56 |
| First nations, Metis, or Inuit | NA | NA, NA | 1 | 0.56 |
| Hispanic or Latino | NA | NA, NA | 0.99 | 0.56 |
| Man | 2.5 | 1.0, 6.90 | 0.06 | 0.34 |
| Non-Binary | NA | NA, NA | 1 | 0.34 |
| Woman | 4.64 | 2.15, 11.22 | 0.0002 | 0.34 |
| Tracking calories or macronutrients | | | | |
| White | 1.96 | 1.01, 3.8 | 0.05 | 0.69 |
| Asian or Pacific Islander | 2.44 | 0.92, 6.5 | 0.07 | 0.69 |
| Black or African American | NA | NA, NA | 0.99 | 0.69 |
| First nations, Metis, or Inuit | NA | NA, NA | 1 | 0.69 |
| Hispanic or Latino | NA | NA, NA | 0.99 | 0.69 |
| Man | 3.95 | 1.52, 10.25 | 0.005 | 0.4 |
| Non-Binary | NA | NA, NA | 1 | 0.4 |
| Woman | 1.8 | 0.94, 3.45 | 0.08 | 0.4 |
| Tracking sleep | | | | |
| White | 3.12 | 1.25, 7.78 | 0.01 | 0.82 |
| Asian or Pacific Islander | 4.19 | 1.27, 13.83 | 0.02 | 0.82 |
| Black or African American | NA | NA, NA | 0.99 | 0.82 |
| First nations, Metis, or Inuit | NA | NA, NA | 1 | 0.82 |
| Hispanic or Latino | NA | NA, NA | 0.99 | 0.82 |
| Man | 2.38 | 0.88, 7.64 | 0.011 | 0.3 |
| Non-Binary | NA | NA, NA | 1 | 0.3 |
| Woman | 5.16 | 2.11, 15.51 | 0.001 | 0.3 |
| Positively Perceived Applications Features | | | | |
| Push notifications with exercise reminders | | | | |
| White | 1.12 | 0.56, 2.36 | 0.75 | 0.58 |
| Asian or Pacific Islander | 2.26 | 1.11, 6.28 | 0.1 | 0.58 |

| | | | | |
|---|---|---|---|---|
| Black or African American | 0.71 | 0.02, 21.1 | 0.83 | 0.58 |
| First nations, Metis, or Inuit | NA | NA, NA | 1 | 0.58 |
| Hispanic or Latino | NA | NA, NA | 1 | 0.58 |
| Man | 0.98 | 0.41, 2.45 | 0.96 | 0.36 |
| Non-Binary | NA | NA, NA | 1 | 0.36 |
| Woman | 1.67 | 0.83, 3.57 | 0.2 | 0.36 |
| Features allowing concrete goal setting | | | | |
| White | 2.26 | 1.15, 4.45 | 0.02 | 0.96 |
| Asian or Pacific Islander | 1.92 | 0.74, 4.99 | 0.18 | 0.96 |
| Black or African American | 1 | 0.05, 19.96 | 1 | 0.96 |
| First nations, Metis, or Inuit | NA | NA, NA | 1 | 0.96 |
| Hispanic or Latino | NA | NA, NA | 0.99 | 0.96 |
| Man | 1.62 | 0.68, 3.84 | 0.28 | 0.66 |
| Non-Binary | NA | NA, NA | 1 | 0.66 |
| Woman | 2.71 | 1.37, 5.34 | 0.004 | 0.66 |
| Ability to visualize progress towards goals | | | | |
| White | 2.19 | 1.13, 4.26 | 0.02 | 0.82 |
| Asian or Pacific Islander | 4 | 1.54, 10.41 | 0.005 | 0.82 |
| Black or African American | 2 | 0.1, 41 | 0.65 | 0.82 |
| First nations, Metis, or Inuit | NA | NA, NA | 1 | 0.82 |
| Hispanic or Latino | NA | NA, NA | 0.99 | 0.82 |
| Man | 2.33 | 0.98, 5.54 | 0.05 | 0.93 |
| Non-Binary | NA | NA, NA | 1 | 0.93 |
| Woman | 2.9 | 1.49, 5.63 | 0.002 | 0.93 |
| Short motivational messages delivered through text | | | | |
| White | 0.62 | 0.21, 1.83 | 0.38 | 0.61 |
| Asian or Pacific Islander | 2.23 | 0.43, 11.5 | 0.34 | 0.61 |
| Black or African American | NA | NA, NA | 0.99 | 0.61 |
| First nations, Metis, or Inuit | NA | NA, NA | 1 | 0.61 |
| Hispanic or Latino | NA | NA, NA | 1 | 0.61 |
| Man | 0.68 | 0.20, 2.70 | 0.6 | 0.35 |
| Non-Binary | NA | NA, NA | 1 | 0.35 |
| Woman | 1.6 | 1.60,7.12 | 0.5 | 0.35 |

Supplemental Table 6. Barriers Associated with Routine Daily or Weekly Physical Activity Application Use

| Characteristic | OR[1] | 95% CI[1] | p-value | p-interaction |
|---|---|---|---|---|
| Lifestyle Barriers to Physical Activity | | | | |
| Not enough time to being more physically active | | | | |
| White | 1.07 | 0.53, 2.13 | 0.85 | 0.72 |
| Asian or Pacific Islander | 1.52 | 0.59, 3.88 | 0.38 | 0.72 |
| Black or African American | NA | NA, NA | 0.99 | 0.72 |
| First nations, Metis, or Inuit | NA | NA, NA | 1 | 0.72 |
| Hispanic or Latino | NA | NA, NA | 0.99 | 0.72 |
| Man | 2.26 | 0.78, 6.55 | 0.13 | 0.34 |
| Non-Binary | NA | NA, NA | 1 | 0.34 |
| Woman | 0.92 | 0.48, 1.75 | 0.79 | 0.34 |
| Not motivated enough to be more physically active | | | | |
| White | 0.3 | 0.15, 0.62 | 0.001 | 0.7 |
| Asian or Pacific Islander | 0.63 | 0.25, 1.56 | 0.32 | 0.7 |
| Black or African American | 1 | 0.05, 20.0 | 1 | 0.7 |
| First nations, Metis, or Inuit | NA | NA, NA | 1 | 0.7 |
| Hispanic or Latino | NA | NA, NA | 0.99 | 0.7 |
| Man | 0.46 | 0.19, 1.09 | 0.08 | 0.99 |
| Non-Binary | NA | NA, NA | 1 | 0.99 |
| Woman | 0.42 | 0.21, 0.82 | 0.01 | 0.99 |
| Limited self-control to follow through on efforts | | | | |
| White | 0.4 | 0.2, 0.78 | 0.007 | 0.79 |
| Asian or Pacific Islander | 0.66 | 0.27, 1.64 | 0.38 | 0.79 |
| Black or African American | 0.5 | 0.02, 10.25 | 0.65 | 0.79 |
| First nations, Metis, or Inuit | NA | NA, NA | 1 | 0.79 |
| Hispanic or Latino | NA | NA, NA | 0.99 | 0.79 |
| Man | 0.4 | 0.17, 0.96 | 0.04 | 0.95 |
| Non-Binary | NA | NA, NA | 1 | 0.95 |
| Woman | 0.48 | 0.25, 0.92 | 0.03 | 0.95 |
| Limited access to recreational facilities | | | | |
| White | 0.47 | 0.22, 1.02 | 0.05 | **0.04** |
| Asian or Pacific Islander | 1.38 | 0.52, 3.87 | 0.53 | **0.04** |
| Black or African American | NA | NA, NA | 1 | **0.04** |
| First nations, Metis, or Inuit | NA | NA, NA | 1 | **0.04** |
| Hispanic or Latino | NA | NA, NA | 1 | **0.04** |
| Man | 1.08 | 0.40,3.25 | 0.89 | 0.3 |
| Non-Binary | 1 | NA, NA | 1 | 0.3 |
| Woman | 0.41 | 0.2, 0.84 | 0.01 | 0.3 |
| Limited energy or rest in day-to-day life | | | | |
| White | 0.53 | 0.28, 1.04 | 0.06 | 0.96 |
| Asian or Pacific Islander | 0.54 | 0.21, 1.35 | 0.19 | 0.96 |
| Black or African American | 0.71 | 0.04, 14.35 | 0.83 | 0.96 |
| First nations, Metis, or Inuit | NA | NA, NA | 1 | 0.96 |

| | | | | |
|---|---|---|---|---|
| Hispanic or Latino | NA | NA, NA | 0.99 | 0.96 |
| Man | 0.36 | 0.15, 0.86 | 0.02 | 0.56 |
| Non-Binary | NA | NA, NA | 1 | 0.56 |
| Woman | 0.65 | 0.34, 1.24 | 0.19 | 0.56 |
| Professional life too demanding | | | | |
| White | 1.48 | 0.76, 2.88 | 0.25 | 0.7 |
| Asian or Pacific Islander | 1.63 | 0.61, 4.39 | 0.33 | 0.7 |
| Black or African American | 0.71 | 0.04, 14.35 | 0.83 | 0.7 |
| First nations, Metis, or Inuit | NA | NA, NA | 1 | 0.7 |
| Hispanic or Latino | NA | NA, NA | 0.99 | 0.7 |
| Man | 2.2 | 0.81, 5.98 | 0.12 | 0.71 |
| Non-Binary | NA | NA, NA | 1 | 0.71 |
| Woman | 1.35 | 0.71, 2.56 | 0.36 | 0.71 |
| Personal or home life too demanding | | | | |
| White | 1.11 | 0.52, 2.36 | 0.79 | 0.47 |
| Asian or Pacific Islander | 2 | 0.69, 5.82 | 0.2 | 0.47 |
| Black or African American | 0.09 | 0, 2.85 | 0.17 | 0.47 |
| First nations, Metis, or Inuit | NA | NA, NA | 1 | 0.47 |
| Hispanic or Latino | NA | NA, NA | 0.99 | 0.47 |
| Man | 0.94 | 0.36, 2.41 | 0.89 | 0.79 |
| Non-Binary | NA | NA, NA | 1 | 0.79 |
| Woman | 1.45 | 0.67, 3.14 | 0.35 | 0.79 |
| Negatively Perceived Application Features | | | | |
| Too many notifications | | | | |
| White | 0.84 | 0.44, 1.62 | 0.6 | 0.21 |
| Asian or Pacific Islander | 0.43 | 0.17, 1.07 | 0.07 | 0.21 |
| Black or African American | NA | NA, NA | 1 | 0.21 |
| First nations, Metis, or Inuit | NA | NA, NA | 1 | 0.21 |
| Hispanic or Latino | NA | NA, NA | 1 | 0.21 |
| Man | 0.76 | 0.32, 1.77 | 0.52 | 0.91 |
| Non-Binary | NA | NA, NA | 1 | 0.91 |
| Woman | 0.6 | 0.32, 1.14 | 0.12 | 0.91 |
| Discouraged when goals unmet | | | | |
| White | 0.49 | 0.25, 0.96 | 0.036 | 0.21 |
| Asian or Pacific Islander | 0.45 | 0.17, 1.16 | 0.1 | 0.21 |
| Black or African American | NA | NA, NA | 1 | 0.21 |
| First nations, Metis, or Inuit | NA | NA, NA | 1 | 0.21 |
| Hispanic or Latino | NA | NA, NA | 1 | 0.21 |
| Man | 0.43 | 0.17, 1.12 | 0.078 | 0.84 |
| Non-Binary | NA | NA, NA | 1 | 0.84 |
| Woman | 0.49 | 0.25, 0.92 | 0.028 | 0.84 |
| Limited personalization for goal setting | | | | |
| White | 0.58 | 0.26, 1.32 | 0.2 | 0.86 |
| Asian or Pacific Islander | 0.37 | 0.13, 1 | 0.05 | 0.86 |
| Black or African American | 0.2 | 0.01, 4.72 | 0.32 | 0.86 |
| First nations, Metis, or Inuit | NA | NA, NA | 1 | 0.86 |

| | | | | |
|---|---|---|---|---|
| Hispanic or Latino | NA | NA, NA | 0.99 | 0.86 |
| Man | 0.52 | 0.18, 1.47 | 0.22 | 0.93 |
| Non-Binary | NA | NA, NA | 1 | 0.93 |
| Woman | 0.41 | 0.2, 0.84 | 0.02 | 0.93 |
| Application too expensive | | | | |
| White | 1.01 | 0.49, 2.19 | 0.97 | 0.31 |
| Asian or Pacific Islander | 0.87 | 0.23, 3.7 | 0.84 | 0.31 |
| Black or African American | NA | NA, NA | 1 | 0.31 |
| First nations, Metis, or Inuit | NA | NA, NA | 1 | 0.31 |
| Hispanic or Latino | NA | NA, NA | 1 | 0.31 |
| Man | 1.02 | 0.34, 3.1 | 0.97 | 1 |
| Non-Binary | NA | NA, NA | 1 | 1 |
| Woman | 1.04 | 0.49, 2.18 | 0.92 | 1 |
| Limited perceived utility | | | | |
| White | 0.42 | 0.19, 0.94 | 0.03 | 0.21 |
| Asian or Pacific Islander | 1.24 | 0.45, 3.70 | 0.68 | 0.21 |
| Black or African American | NA | NA, NA | 1 | 0.21 |
| First nations, Metis, or Inuit | NA | NA, NA | 1 | 0.21 |
| Hispanic or Latino | NA | NA, NA | 1 | 0.21 |
| Man | 0.83 | 0.31, 2.35 | 0.71 | 0.58 |
| Non-Binary | NA | NA, NA | 1 | 0.58 |
| Woman | 0.42 | 0.19, 0.96 | 0.04 | 0.58 |
| Insufficient number of features | | | | |
| White | z | 0.22, 0.87 | 0.02 | 0.09 |
| Asian or Pacific Islander | 0.43 | 0.17, 1.08 | 0.07 | 0.09 |
| Black or African American | NA | NA, NA | 1 | 0.09 |
| First nations, Metis, or Inuit | NA | NA, NA | 1 | 0.09 |
| Hispanic or Latino | NA | NA, NA | 1 | 0.09 |
| Man | 0.5 | 0.21, 1.17 | 0.11 | 0.96 |
| Non-Binary | NA | NA, NA | 1 | 0.96 |
| Woman | 0.58 | 0.3, 1.12 | 0.1 | 0.96 |
| Application did not meet accessibility standards | | | | |
| White | 0.48 | 0.09, 3.57 | 0.41 | 0.9 |
| Asian or Pacific Islander | 0.57 | 0.07, 4.97 | 0.59 | 0.9 |
| Black or African American | NA | NA, NA | 1 | 0.9 |
| First nations, Metis, or Inuit | NA | NA, NA | 1 | 0.9 |
| Hispanic or Latino | NA | NA, NA | 1 | 0.9 |
| Man | 0.31 | 0.01, 8.02 | 0.41 | 0.77 |
| Non-Binary | NA | NA, NA | 1 | 0.77 |
| Woman | 0.5 | 0.12, 2.50 | 0.35 | 0.77 |
| Application required expensive peripheral devices | | | | |
| White | 0.7 | 0.30, 1.78 | 0.43 | 0.87 |
| Asian or Pacific Islander | 0.5 | 0.17, 1.46 | 0.2 | 0.87 |
| Black or African American | NA | NA, NA | 1 | 0.87 |
| First nations, Metis, or Inuit | NA | NA, NA | 1 | 0.87 |
| Hispanic or Latino | NA | NA, NA | 1 | 0.87 |

| | | | | |
|---|---|---|---|---|
| Man | 0.47 | 0.17, 1.41 | 0.16 | 0.62 |
| Non-Binary | NA | NA, NA | 1 | 0.62 |
| Woman | 0.66 | 0.30, 1.56 | 0.32 | 0.62 |
| Application was difficult to use | | | | |
| White | 1.24 | 0.19, 24.1 | 0.84 | 0.2 |
| Asian or Pacific Islander | NA | NA, NA | 1 | 0.2 |
| Black or African American | NA | NA, NA | 1 | 0.2 |
| First nations, Metis, or Inuit | NA | NA, NA | 1 | 0.2 |
| Hispanic or Latino | NA | NA, NA | 1 | 0.2 |
| Man | 0.95 | 0.12, 19.7 | 0.97 | 0.18 |
| Non-Binary | NA | NA, NA | 1 | 0.18 |
| Woman | NA | NA, NA | 1 | 0.18 |
| Limited aesthetic appeal | | | | |
| White | 1.13 | 0.44, 2.93 | 0.8 | 0.69 |
| Asian or Pacific Islander | 0.88 | 0.33, 2.39 | 0.81 | 0.69 |
| Black or African American | NA | NA, NA | 0.99 | 0.69 |
| First nations, Metis, or Inuit | NA | NA, NA | 1 | 0.69 |
| Hispanic or Latino | NA | NA, NA | 0.99 | 0.69 |
| Man | 0.88 | 0.33, 2.37 | 0.8 | 0.98 |
| Non-Binary | NA | NA, NA | 1 | 0.98 |
| Woman | 1 | 0.42, 2.38 | 0.99 | 0.98 |
| Application had data privacy concerns | | | | |
| White | 0.58 | 0.22, 1.72 | 0.3 | 0.37 |
| Asian or Pacific Islander | 1.07 | 0.33, 3.82 | 0.91 | 0.37 |
| Black or African American | NA | NA, NA | 1 | 0.37 |
| First nations, Metis, or Inuit | NA | NA, NA | 1 | 0.37 |
| Hispanic or Latino | NA | NA, NA | 1 | 0.37 |
| Man | 0.86 | 0.25, 2.94 | 0.81 | 0.99 |
| Non-Binary | NA | NA, NA | 1 | 0.99 |
| Woman | 0.77 | 0.3, 1.96 | 0.58 | 0.99 |
| Application was not evidence based | | | | |
| White | 2.65 | 0.74, 17.0 | 0.2 | **0.03** |
| Asian or Pacific Islander | 0.36 | 0.10, 1.24 | 0.11 | **0.03** |
| Black or African American | NA | NA, NA | 1 | **0.03** |
| First nations, Metis, or Inuit | NA | NA, NA | 1 | **0.03** |
| Hispanic or Latino | NA | NA, NA | 1 | **0.03** |
| Man | 0.72 | 0.19, 3.53 | 0.65 | 0.98 |
| Non-Binary | NA | NA, NA | 1 | 0.98 |
| Woman | 0.86 | 0.33, 2.50 | 0.77 | 0.98 |

# Health and Well-being Perception and Engagement with Exercise Applications
## Instructions & Consent Page

- Thank you for your interest in participating in this study conducted by The Decision Lab. We are a socially-conscious applied research firm that uses behavioural science to improve outcomes for everybody in society.
- The purpose of this study is to better understand how people engage with exercise applications for the benefit of their health and wellbeing
- The study is administered as a series of questions and will take approximately 10 minutes.
- Your participation in this study is entirely voluntary. By participating, you consent to anonymously contribute your responses. If at any point you wish to discontinue for any reason whatsoever, you are free to do so.
- If you stop part way through the study, any data that you have contributed will be deleted.
- All data collected will be stripped of any personal identifiers and will be stored on an access-restricted server until the completion of the study.
- Any scientific findings that arise as a result of this study will be published on The Decision Lab website (https://www.thedecisionlab.com/) as well as other venues to be determined.
- If you have any questions or concerns, please contact our ethics officer at info@thedecisionlab.com

# Health and Well-being Perception and Engagement with Exercise Applications
## General Wellbeing

These questions will help us understand your perception of your general health and wellbeing, as well the habits that underlie it.

* 1. For each of the questions or statements please select the response that best describes your thoughts or feelings.

| | Very Poor | Poor | Average | Good | Very Good |
|---|---|---|---|---|---|
| How would you describe your personal health? | ◯ | ◯ | ◯ | ◯ | ◯ |
| How would you describe your physical fitness? | ◯ | ◯ | ◯ | ◯ | ◯ |
| How would you describe your emotional health and wellbeing? | ◯ | ◯ | ◯ | ◯ | ◯ |

* 2. How important is your physical health and wellbeing?

| Very unimportant | Unimportant | Neutral | Important | Very important |
|---|---|---|---|---|
| ◯ | ◯ | ◯ | ◯ | ◯ |

## Health and Well-being Perception and Engagement with Exercise Applications

### General Wellbeing

* 3. Which of the following aspects of your physical health and wellbeing are important to you? Please select all that apply.

☐ Losing weight

☐ Gaining weight

☐ Being more active

☐ Improving mobility

☐ Improving eating habits

☐ Lowering cholesterol

☐ Improving sleep

☐ Gaining muscle

☐ Improving mental wellbeing

☐ Other (please specify)

## Health and Well-being Perception and Engagement with Exercise Applications

### General Wellbeing

* 4. I would like to improve my physical health or wellness

| Strongly disagree | Disagree | Neutral | Agree | Strongly Agree |
|---|---|---|---|---|
| ◯ | ◯ | ◯ | ◯ | ◯ |

## Health and Well-being Perception and Engagement with Exercise Applications

### General Wellbeing

* 5. Which of the following areas of your physical health or wellness are you most interested in improving? Please select all that apply.

- ☐ Losing weight
- ☐ Gaining weight
- ☐ Being more active
- ☐ Improving mobility
- ☐ Improving eating habits
- ☐ Lowering cholesterol
- ☐ Improving sleep
- ☐ Gaining muscle
- ☐ Improving mental wellbeing
- ☐ Other (please specify)

## Health and Well-being Perception and Engagement with Exercise Applications

### Barriers to Physical Activity

These questions will help us to understand barriers that you may face towards improving your physical health and wellbeing.

* 6. For each of the questions or statements please select the response that best

describes your thoughts or feelings.

| | Strongly disagree | Disagree | Neutral | Agree | Strongly Agree |
|---|---|---|---|---|---|
| I am interested in increasing my daily levels of physical activity | ○ | ○ | ○ | ○ | ○ |
| I don’t have enough time to put towards being more physically active | ○ | ○ | ○ | ○ | ○ |
| I am not motivated enough to be more physically active | ○ | ○ | ○ | ○ | ○ |
| I don’t have enough self control to follow through on efforts to be more physically active | ○ | ○ | ○ | ○ | ○ |
| I do not have easy access to facilities (gyms, parks, pools, etc.), and this makes it harder to be more physically active | ○ | ○ | ○ | ○ | ○ |
| I’m generally too tired or poorly rested to be more physically active | ○ | ○ | ○ | ○ | ○ |
| My professional life makes it difficult to be more physically active | ○ | ○ | ○ | ○ | ○ |
| My personal or home life makes it difficult to be more physically active | ○ | ○ | ○ | ○ | ○ |
| My family would be supportive of | | | | | |

| | | | | | |
|---|---|---|---|---|---|
| any efforts I make to become more physically active | ◯ | ◯ | ◯ | ◯ | ◯ |
| My friends would be supportive of any efforts I make to become more physically active | ◯ | ◯ | ◯ | ◯ | ◯ |

## Health and Well-being Perception and Engagement with Exercise Applications

### Past efforts to improve physical activity

These questions will help us to understand strategies that have worked for you in efforts to improve your physical health and wellbeing.  For each of the questions or statements please select the response that best describes your thoughts or feelings.

* 7. Have you, in the past, encountered strategies that enabled you to become more physically active or otherwise improve your physical health and wellbeing?

◯ Yes

◯ No


## Health and Well-being Perception and Engagement with Exercise Applications

### Past efforts to improve physical activity


* 8. For each of the questions or statements please select the response that best describes your thoughts or feelings.

| | Strongly disagree | Disagree | Neutral | Agree | Strongly Agree |
|---|---|---|---|---|---|
| Forming consistent habits made it easier for me to become more physically active or otherwise improve my physical health and wellbeing | ◯ | ◯ | ◯ | ◯ | ◯ |

| | | | | | |
|---|---|---|---|---|---|
| External motivation from a coach or trainer made it easier for me to become more physically active or otherwise improve my physical health and wellbeing | ◯ | ◯ | ◯ | ◯ | ◯ |
| External motivation from a significant other family member, friend, or other loved one made it easier for me to become more physically active or otherwise improve my physical health and wellbeing | ◯ | ◯ | ◯ | ◯ | ◯ |
| Paid training courses or programs made it easier for me to become more physically active or otherwise improve my physical health and wellbeing | ◯ | ◯ | ◯ | ◯ | ◯ |
| Mobile applications or other digital products made it easier for me to become more physically active or otherwise improve my physical health and wellbeing | ◯ | ◯ | ◯ | ◯ | ◯ |
| I generally found that increasing physical activity or otherwise improving my physical health or wellbeing took | ◯ | ◯ | ◯ | ◯ | ◯ |

less time than I
expected it to

* 9. Increasing the extent to which I prioritize my physical health was important towards my efforts to become more physically active or otherwise improve my physical health or wellbeing

| Strongly disagree | Disagree | Neutral | Agree | Strongly Agree |
| --- | --- | --- | --- | --- |
| ◯ | ◯ | ◯ | ◯ | ◯ |

## Health and Well-being Perception and Engagement with Exercise Applications

### Past efforts to improve physical activity

10. Please explain any strategies that you found successful in increasing the extent to which you prioritized your physical health.

## Health and Well-being Perception and Engagement with Exercise Applications

### Health and Fitness Apps

These questions will help us to understand your experience using health and fitness apps on your phone.  For each of the questions or statements please select the response that best describes your thoughts or feelings.

* 11. Do you evaluate the credibility and quality of an app before you download it?

◯ Yes

◯ No

## Health and Well-being Perception and Engagement with Exercise Applications

## Health and Fitness Apps

* 12. How do you evaluate the quality and credibility of an app?

- ☐ Online Reviews
- ☐ Referrals from friends, family, or peers
- ☐ Brand recognition (e.g. whether i am familiar with the company who developed the app)
- ☐ Referrals from online personalities (e.g. YouTube videos and influencers)
- ☐ Ratings in the app store
- ☐ Other (please specify)

# Health and Well-being Perception and Engagement with Exercise Applications

## Health and Fitness Apps

* 13. Do you currently use any health or fitness apps on your phone?

- ○ Yes
- ○ No

# Health and Well-being Perception and Engagement with Exercise Applications

## Health and Fitness Apps

* 14. Which health and fitness apps do you use? Please select all that apply

- [ ] MyFitness Pal
- [ ] Fitbit
- [ ] Samsung Health
- [ ] Mi Fit
- [ ] Other (please specify)

* 15. How frequently do you use these applications?

- ○ Daily
- ○ Weekly
- ○ Monthly
- ○ Whenever I am reminded to do so
- ○ Other (please specify)

* 16. Mobile applications help me track progress towards my health and fitness goals

| Strongly disagree | Disagree | Neutral | Agree | Strongly Agree |
|---|---|---|---|---|
| ○ | ○ | ○ | ○ | ○ |

## Health and Well-being Perception and Engagement with Exercise Applications

### Health and Fitness Apps

* 17. Please indicate what type of goals you track through mobile health and fitness applications. Please select all that apply

- [ ] Steps
- [ ] Calories or macronutrients
- [ ] Time spent exercising
- [ ] Sleep
- [ ] Heart rate
- [ ] Other (please specify)

## Health and Well-being Perception and Engagement with Exercise Applications

Health and Fitness Apps

* 18. Mobile health and fitness applications help me find and maintain motivation to improve my physical health and wellbeing

| Strongly disagree | Disagree | Neutral | Agree | Strongly Agree |
|---|---|---|---|---|
| ○ | ○ | ○ | ○ | ○ |

## Health and Well-being Perception and Engagement with Exercise Applications

Health and Fitness Apps

* 19. Please indicate what app-based strategies have helped you find and maintain motivation. Please select all that apply

- ☐ Push notifications reminding me to exercise
- ☐ Setting concrete goals
- ☐ Visualizing my progress towards goals
- ☐ Short motivational messages delivered through text
- ☐ Motivational voice recordings from athletes or prominent fitness figures
- ☐ Other (please specify)

## Health and Well-being Perception and Engagement with Exercise Applications

### Health and Fitness Apps

* 20. Please indicate what app-based strategies could possibly help you find and maintain motivation. Please select all that apply

- ☐ Push notifications reminding me to exercise
- ☐ Setting concrete goals
- ☐ Visualizing my progress towards goals
- ☐ Calories or macronutrients
- ☐ Short motivational messages delivered through text
- ☐ Motivational voice recordings from athletes or prominent fitness figures
- ☐ Other (please specify)

## Health and Well-being Perception and Engagement with Exercise Applications

### Health and Fitness Apps

* 21. Mobile health and fitness applications help keep me accountable for my health and fitness goals

| Strongly disagree | Disagree | Neutral | Agree | Strongly Agree |
| --- | --- | --- | --- | --- |
| ◯ | ◯ | ◯ | ◯ | ◯ |

## Health and Well-being Perception and Engagement with Exercise Applications

### Health and Fitness Apps

* 22. Mobile health and fitness applications are a more cost effective solution than hiring a personal trainer or coach

| Strongly disagree | Disagree | Neutral | Agree | Strongly Agree |
| --- | --- | --- | --- | --- |
| ◯ | ◯ | ◯ | ◯ | ◯ |

## Health and Well-being Perception and Engagement with Exercise Applications

### Health and Fitness Apps

* 23. Mobile health and fitness applications are convenient to use

| Strongly disagree | Disagree | Neutral | Agree | Strongly Agree |
| --- | --- | --- | --- | --- |
| ◯ | ◯ | ◯ | ◯ | ◯ |

## Health and Well-being Perception and Engagement with Exercise Applications

### Health and Fitness Apps

24. Please explain what specific aspects of these applications present an inconvenience.

## Health and Well-being Perception and Engagement with Exercise Applications

### Health and Fitness Apps

* 25. Please indicate which, if any, of the following adequately describe any issues you have had with mobile health and fitness applications in the past. Please select all that apply.

- [ ] Too many notifications
- [ ] Made me feel bad when i fell behind or missed goals
- [ ] I wasn't able to set goals that were useful to me - it was too rigid
- [ ] It was too expensive
- [ ] It wasn't that useful
- [ ] The free version didn't have enough features
- [ ] The app didn't meet accessibility standards
- [ ] It required expensive technology to use (e.g. a heart rate monitor or other wearable device)
- [ ] They were too hard to use
- [ ] My phone can't run them effectively
- [ ] Bad or ugly aesthetic design
- [ ] I was concerned about my data privacy
- [ ] There was no evidence that it would be effective
- [ ] None of the above
- [ ] Other (please specify)


## Health and Well-being Perception and Engagement with Exercise Applications

### Health and Fitness Apps

These questions will help us to understand your experience or lack thereof with using health and fitness apps on your phone.  For each of the questions or statements please select the response that best describes your thoughts or feelings.

* 26. Have you used health and fitness apps on your phone at any point in the past?

- ◯ Yes
- ◯ No

## Health and Well-being Perception and Engagement with Exercise Applications

### Health and Fitness Apps

* 27. Which of the following factors contributed to your decision to stop using them? Please select all that apply.

- ☐ Too many notifications
- ☐ Made me feel bad when I fell behind or missed goals
- ☐ I wasn't able to set goals that were useful to me - it was too rigid
- ☐ It was too expensive
- ☐ It wasn't that useful
- ☐ The free version didn't have enough features
- ☐ The app didn't meet accessibility standards
- ☐ It required expensive technology to use (e.g. a heart rate monitor or other wearable device)
- ☐ They were too hard to use
- ☐ My phone can't run them effectively
- ☐ Bad or ugly aesthetic design
- ☐ I was concerned about my data privacy
- ☐ There was no evidence that it would be effective
- ☐ No particular reason
- ☐ Other (please specify)

## Health and Well-being Perception and Engagement with Exercise

## Applications
### Health and Fitness Apps

* 28. Why do you not feel the need to use health or fitness apps? Please select all that apply

- [ ] I don't care that much about improving my physical health or fitness
- [ ] I'm worried they might make me feel guilty or lazy
- [ ] They are expensive
- [ ] I don't know what features they offer
- [ ] I don't know of any good apps
- [ ] My approach to physical health is unique and existing apps are too generalized
- [ ] I have accessibility requirements that aren't met by most apps
- [ ] They require expensive technology to use (e.g. a heart rate monitor or other wearable device)
- [ ] No particular reason
- [ ] Other (please specify)

## Health and Well-being Perception and Engagement with Exercise Applications
### Health and Fitness Apps

* 29. I am interested in tracking progress towards my health and fitness goals

| Strongly disagree | Disagree | Neutral | Agree | Strongly Agree |
|---|---|---|---|---|
| ○ | ○ | ○ | ○ | ○ |

## Health and Well-being Perception and Engagement with Exercise Applications
### Health and Fitness Apps

* 30. Please indicate what type of goals you might be interested in tracking through mobile health and fitness applications. Please select all that apply

- [ ] Steps
- [ ] Time spent exercising
- [ ] Heart rate
- [ ] Other (please specify)
- [ ] Calories or macronutrients
- [ ] Sleep

## Health and Well-being Perception and Engagement with Exercise Applications

### Health and Fitness Apps

* 31. I am interested in improving my motivation to exercise or otherwise improve my physical health and wellbeing

| Strongly disagree | Disagree | Neutral | Agree | Strongly Agree |
|---|---|---|---|---|
| ○ | ○ | ○ | ○ | ○ |

## Health and Well-being Perception and Engagement with Exercise Applications

### Health and Fitness Apps

* 32. Please indicate which app-based strategies might help you find and maintain motivation. Please select all that apply

- ☐ Push notifications reminding me to exercise
- ☐ Setting concrete goals
- ☐ Visualizing my progress towards goals
- ☐ Short motivational messages delivered through text
- ☐ Motivational voice recordings from athletes or prominent fitness figures
- ☐ Other (please specify)

## Health and Well-being Perception and Engagement with Exercise Applications

### Privacy and Security

These questions will help us to understand your attitudes and concerns towards your data privacy while using health and fitness apps.  For each of the questions or statements please select the response that best describes your thoughts or feelings.

* 33. I am concerned about the privacy and security of my personal information when using a health and fitness app or mobile apps more broadly

| Strongly disagree | Disagree | Neutral | Agree | Strongly Agree |
|---|---|---|---|---|
| ◯ | ◯ | ◯ | ◯ | ◯ |

## Health and Well-being Perception and Engagement with Exercise Applications

### Privacy and Security

* 34. Which of the following security features would make you more comfortable with the safety and security of your personal information? Please select all that apply.

- [ ] A privacy policy
- [ ] A clear privacy policy with no complicated legal language
- [ ] Blockchain technology
- [ ] Local storage of information
- [ ] Other (please specify)
- [ ] Transparency regarding how data is being collected, used, or shared
- [ ] Option to opt out of sharing data
- [ ] I’m not sure

## Health and Well-being Perception and Engagement with Exercise Applications

### Privacy and Security

* 35. How would you feel if a physical health and wellness application collected your anonymized health or fitness information explicitly for academic research purposes? (For clarity, “anonymized” entails the removal of any personally identifiable information like name, email address, date of birth, etc.)

| Very uncomfortable | Uncomfortable | Neutral | Comfortable | Very comfortable |
|---|---|---|---|---|
| ○ | ○ | ○ | ○ | ○ |

* 36. How might this affect your attitudes towards using the application? Please select all that apply

- [ ] I would refuse to use it outright
- [ ] I would be uncomfortable with using it
- [ ] I would use it more
- [ ] It would depend on what data is being shared
- [ ] It would depend on the nature of the organization in question

## Health and Well-being Perception and Engagement with Exercise

## Applications
### Privacy and Security

* 37. How would you feel if, instead of an academic entity, anonymized data was to be shared with a private entity

| Very uncomfortable | Uncomfortable | Neutral | Comfortable | Very comfortable |
|---|---|---|---|---|
| ◯ | ◯ | ◯ | ◯ | ◯ |

* 38. How might this affect your attitudes towards using the application? Please select all that apply

- ☐ I would refuse to use it outright
- ☐ I would be uncomfortable with using it
- ☐ I would use it more
- ☐ It would depend on what data is being shared
- ☐ It would depend on the nature of the organization in question

## Health and Well-being Perception and Engagement with Exercise Applications
### Privacy and Security

* 39. How would you feel if the app were to share anonymized data with academic research entities in exchange for compensation?

| Very uncomfortable | Uncomfortable | Neutral | Comfortable | Very comfortable |
|---|---|---|---|---|
| ◯ | ◯ | ◯ | ◯ | ◯ |

* 40. How might this affect your attitudes towards using the application? Please select all that apply

- ☐ I would refuse to use it outright
- ☐ I would be uncomfortable with using it
- ☐ I would use it more
- ☐ It would depend on what data is being shared
- ☐ It would depend on the nature of the organization in question

## Health and Well-being Perception and Engagement with Exercise Applications

### Privacy and Security

* 41. How would you feel if the app were to share anonymized data with private entities in exchange for compensation?

| Very uncomfortable | Uncomfortable | Neutral | Comfortable | Very comfortable |
| --- | --- | --- | --- | --- |
| ◯ | ◯ | ◯ | ◯ | ◯ |

* 42. How might this affect your attitudes towards using the application? Please select all that apply

- ☐ I would refuse to use it outright
- ☐ I would be uncomfortable with using it
- ☐ I would use it more
- ☐ It would depend on what data is being shared
- ☐ It would depend on the nature of the organization in question

## Health and Well-being Perception and Engagement with Exercise Applications

### Cost and benefits

These questions will help us to understand your attitudes and concerns towards the cost of using health and fitness apps. For each of the questions or statements please select the response that best describes your thoughts or feelings.

* 43. Cost is a significant factor that I consider when deciding whether or not to download a mobile application

| Strongly disagree | Disagree | Neutral | Agree | Strongly Agree |
| --- | --- | --- | --- | --- |
| ◯ | ◯ | ◯ | ◯ | ◯ |

## Health and Well-being Perception and Engagement with Exercise Applications

### Cost and benefits

* 44. Have you purchased private insurance or do you have access to it through some other means (federal program, job benefit, etc.)

◯ Yes

◯ No

## Health and Well-being Perception and Engagement with Exercise Applications

### Cost and benefits

* 45. If my insurance costs would be reduced under the condition that I use a health or wellness application I would be significantly more likely to do so

| Strongly disagree | Disagree | Neutral | Agree | Strongly Agree |
| --- | --- | --- | --- | --- |
| ◯ | ◯ | ◯ | ◯ | ◯ |

## Health and Well-being Perception and Engagement with Exercise Applications

### Cost and benefits

* 46. Cash or cash-equivalent incentives (e.g. gift cards) delivered in exchange for using an app would motivate me to engage regularly with a mobile application?

| Strongly disagree | Disagree | Neutral | Agree | Strongly Agree |
| --- | --- | --- | --- | --- |
| ◯ | ◯ | ◯ | ◯ | ◯ |

## Health and Well-being Perception and Engagement with Exercise Applications

### Demographics

* 47. What province/territory do you live in?

- British Columbia
- Alberta
- Saskatchewan
- Manitoba
- Ontario
- Quebec
- New Brunswick
- Nova Scotia
- Prince Edward Island
- Newfoundland and Labrador
- Yukon
- Northwest Territories
- Nunavut
- Other (please specify)

* 48. How would you describe the area in which you live?

- City/Urban
- Suburban
- Rural

* 49. What is your gender?

- Woman
- Man
- Non-Binary
- Prefer not to answer

* 50. What is the highest level of education you've completed?

- ◯ None
- ◯ Elementary Scales (K-5; 6-8; 9-12)
- ◯ High school diploma or equivalent
- ◯ Some college, no degree
- ◯ Associate's degree, occupational or academic
- ◯ Bachelor's degree
- ◯ Master's Degree
- ◯ Professional Degree (e.g.: Medicine, Law, Dentistry, etc.)
- ◯ Doctoral Degree

* 51. What is your annual household income (before taxes) as of **August 2021**?

- ◯ Under $15,000
- ◯ $15,000 to $24,999
- ◯ $25,000 to $34,999
- ◯ $35,000 to $49,999
- ◯ $50,000 to $74,999
- ◯ $75,000 to $99,999
- ◯ $100,000 to $149,999
- ◯ $150,000 to $199,999
- ◯ $200,000 or more

* 52. What is your employment status as of **August 2021**?

- ◯ Full-time employment
- ◯ Part-time employment
- ◯ Unemployed, job searcher/seeker
- ◯ Unemployed, left job
- ◯ Unemployed, temporary layoff
- ◯ Unemployed, lost job
- ◯ Not in labor force

* 53. What is your marital status as of **August 2021**?

- ◯ Single (Never Married)
- ◯ Living with my partner (not married)
- ◯ Married
- ◯ Separated
- ◯ Divorced
- ◯ Widowed

* 54. How many people live in your household?

* 55. What is your ethnicity? (Select all that apply)

- [ ] White
- [ ] First nations, Metis, or Inuit
- [ ] Hispanic or Latino
- [ ] Asian or Pacific Islander
- [ ] Black or African American

* 56. Do you have a disability?

- ( ) Yes
- ( ) No

* 57. What is your prolific ID

## Health and Well-being Perception and Engagement with Exercise Applications

Thank you for participating

Click this link to return to prolific

https://app.prolific.co/submissions/complete?cc=2EBA32CF
